# HIPPIE: a generative model for electrophysiological analysis across species, technologies, and modalities

**DOI:** 10.1038/s41467-026-76939-w

**Published:** 2026-08-21

**Authors:** Jesus Gonzalez-Ferrer, Julian Lehrer, Bruno Alvarez-Esteban, Avelina Moreno-Ochando, Hunter E. Schweiger, Jinghui Geng, Luiz F. S. Eugenio dos Santos, Sebastian Hernandez, Francisco Reyes, Jess L. Sevetson, Aidan Schneider, Sofie R. Salama, Mircea Teodorescu, David Haussler, Mohammed A. Mostajo-Radji

**Affiliations:** 1 Genomics Institute, University of California Santa Cruz, Santa Cruz, CA USA; 2 Live Cell Biotechnology Discovery Lab, University of California Santa Cruz, Santa Cruz, CA USA; 3 Department of Biomolecular Engineering, University of California Santa Cruz, Santa Cruz, CA USA; 4 Department of Molecular, Cellular and Developmental Biology, University of California Santa Cruz, Santa Cruz, CA USA; 5 Department of Electrical and Computer Engineering, University of California Santa Cruz, Santa Cruz, CA USA; 6 Biotechnology Program, Berkeley City College, Berkeley, CA USA; 7 Department of Statistics & Data Science, Yale University, New Haven, CT USA; 8 Wu Tsai Institute, Yale University, New Haven, CT USA

**Keywords:** Neurophysiology, Data processing

## Abstract

Neuronal classification from extracellular electrophysiological recordings is challenging due to intrinsic waveform variability, noise, and technical differences across experiments, technologies, and species. We introduce HIPPIE (High-dimensional Interpretation of Physiological Patterns In Intercellular Electrophysiology), a deep learning framework that combines self-supervised pretraining on unlabeled datasets with supervised fine-tuning to classify neurons from extracellular recordings. Using conditional convolutional joint autoencoders, HIPPIE learns technology-adjusted representations of waveforms and spiking dynamics. Here we show, across mouse, rat, and macaque recordings, that HIPPIE classifies cell types competitively with existing methods while additionally supporting generative analyses that discriminative models cannot perform, including counterfactual decoding of electrophysiological signals under changed experimental conditioning, cross-species latent interpolation, and a cross-modal analysis revealing that spike-timing modalities and waveform morphology encode largely independent dimensions of neuronal identity. HIPPIE is available as both a Python package and a coding-free web application, providing a unified framework for multimodal neuronal classification across technologies, experimental conditions, and species.

## Abstract (web summary)

This presents HIPPIE, a deep-learning model that classifies neuron types from electrophysiological recordings across mice, rats and macaques, and can generate and translate signals between species or recording technologies.

## Introduction

Advances in high-throughput sequencing have enabled extensive single-cell gene expression profiling, resolving cellular diversity at scale^1,2^. However, transcriptional profiles do not capture functional properties such as electrophysiology, which are critical for understanding how neurons operate within their native circuits^3^.

To date, most electrophysiology-based cell-type classifications rely on low-throughput methods such as whole-cell patch clamping^4,5^. However, several high-throughput technologies have recently emerged, including planar high-density microelectrode arrays^6–9^, flexible three-dimensional microelectrode array baskets^10,11^, and high-density probes such as Neuropixels^12,13^. These platforms enable extracellular recordings at scale, allowing simultaneous monitoring of multiple neurons across regions and facilitating the study of network-level dynamics. High-throughput electrophysiology has traditionally distinguished broad cell-type categories using scalar measures such as action potential width, separating putative excitatory broad-spiking neurons from inhibitory narrow-spiking neurons^14^. In some cases, combining these approaches with genetic tools like optotagging^15,16^ has enabled the identification of specific neuronal subtypes within large-scale recordings.

Efforts to classify extracellular electrophysiological data have diverged into supervised and unsupervised paradigms. Supervised methods such as LOLCAT^17^ achieve high performance on labeled data but are constrained by the scarcity of annotated datasets. Unsupervised approaches, including non-linear dimensionality reduction for clustering extracellular waveforms^18^ or spiking patterns^19,20^, leverage unlabeled data but typically operate on single modalities. Multimodal integration has become a priority: PhysMAP^21^ adapts single-cell genomics strategies^22^ to electrophysiology via UMAP and weighted nearest neighbors, while VAE frameworks^23^ unify modalities through shared latent spaces. Self-supervised contrastive models such as NEMO^24^ align embeddings across modalities without reconstruction objectives, achieving high classification accuracy on labeled benchmarks. These methods have advanced electrophysiological cell-type classification; however, they do not combine, in a single framework, conditioning on experimental metadata for cross-technology and cross-species transfer, arbitrary modality extension, and generative analysis of the learned representation space.

Conditional VAEs (cVAEs) complement discriminative approaches by balancing classification objectives with generative flexibility^25^; their modular encoder structure accommodates additional input modalities without architectural changes. Unlike standard VAEs, cVAEs condition latent representations on auxiliary variables such as experimental parameters, enabling targeted feature extraction and cross-condition synthesis^25^. Applied to electrophysiology, cVAEs can decompose shared and modality-specific neuronal traits without requiring spatial priors, making them particularly suitable for in vitro models lacking anatomical context. By explicitly modeling conditional dependencies such as brain region or recording technology, cVAEs integrate spike waveforms, temporal dynamics, and metadata into a unified latent space that supports both classification and generation.

We introduce HIPPIE, which implements this framework for extracellular electrophysiology, conditioning on recording technology, species, and brain region to isolate biological signals from technical noise and synthesize recordings under changed experimental conditions. This enables capabilities that discriminative baselines cannot provide: cross-modal imputation of missing recording modalities and counterfactual decoding under altered conditioning. Validated across mouse, rat, and macaque recordings, HIPPIE is competitive with existing classification methods while also supporting generative analysis.

## Results

### The HIPPIE pipeline

**Fig. 1:**
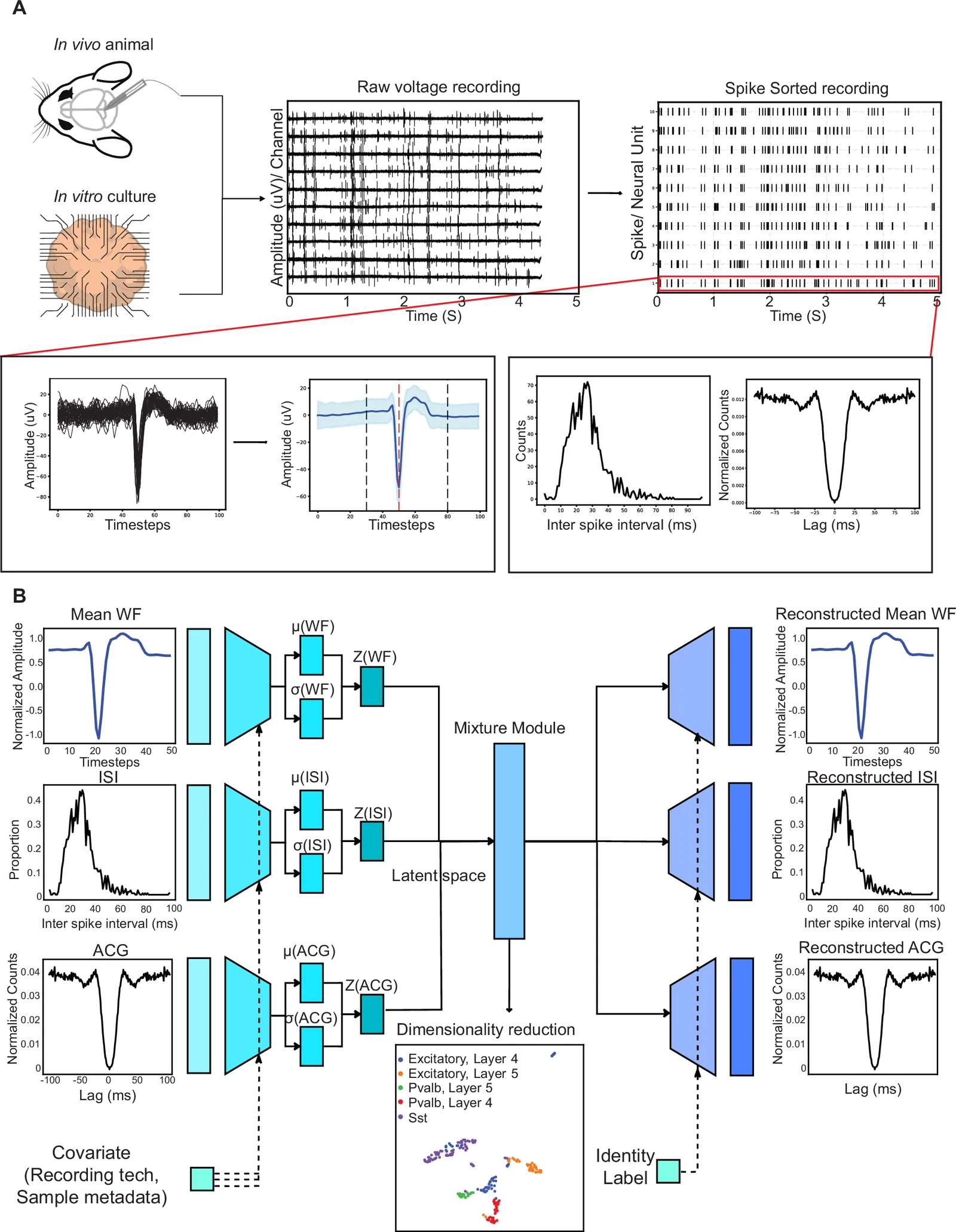
The HIPPIE pipeline. **A** Data from in vivo or in vitro experiments are spike sorted. For each unit, spike timings are collected from the reconstructed spike train and used to compute the interspike interval distribution; waveforms are extracted as a 2.5 ms window centered on the action potential peak from the corresponding raw voltage recordings. The mouse diagram was adapted from Luigi Petrucco’s SciDraw illustration (2026; Mouse head schema; https://scidraw.io/drawing/mouse-head-schema-51ed02be). The organoid diagram was adapted from the organoid icon by Marcel Tisch in Bioicons. **B** One-dimensional vectors representing the mean waveform (WF), interspike interval (ISI) distribution, and autocorrelogram (ACG) are fed into conditional VAEs trained in parallel, and a mixture module builds a joint representation of the modalities in the embedding space. Pvalb parvalbumin-positive interneuron, Sst somatostatin-positive interneuron.

The HIPPIE architecture comprises parallel cVAEs built from 1dResNet^26^ (one-dimensional residual network) encoder and decoder modules (Fig. 1).

The HIPPIE pipeline takes as input spike-sorted data from extracellular electrophysiology recordings. From these, the average waveform, autocorrelogram (ACG), and interspike interval (ISI) distribution are calculated as one-dimensional vectors. These vectors are appended with a categorical label representing the recording technology used in the experiment. During training, the cell type or brain structure are also appended so the model can learn biologically relevant representations. This combined input is then provided to the cVAEs.

The average waveform component of the input is calculated separately for each unit, combining data from a sample of 100–500 spikes from that unit. The ISI distributions are computed from the spike train list using 1-ms bins spanning a total of 100 ms. The ACG is also calculated from the spike train list using 1-ms bins spanning a total of 200 ms.

The pipeline^27^ deploys a spike sorter with template matching, which yields relatively low waveform variability across spikes. This approach allows reliable spike representations even for low firing rate neurons with around 100 spikes. Given an average firing rate of 2 Hz, a 10-min recording would yield ~1200 spikes, from which the 500-spike sample provides a representative subset.

The length of the extracted waveform is 2.5 ms, corresponding to 50 data points when sampled at 20 kHz. The software accommodates higher sampling rates by interpolating down to 50 points. A 2.5 ms time window captures most of the action potential duration in most neurons. If the algorithm were to be adapted for other electrophysiologically active cells, the time window could be adjusted accordingly. The preprocessing pipeline used to obtain these processed features from raw voltage recordings is described in more detail in “Methods”. We focused on average waveform, ACG, and ISI distribution because these features correlate strongly with cell type and brain region^18,28^, but the framework can also be extended with additional measurements such as anatomic information or connectivity-related features^29^.

The outputs from the encoding layers of different modalities are integrated using a mixture module. In this work, the mixture module is implemented as a multilayer perceptron (MLP)^30^, a feedforward neural network that combines information from multiple sources, which takes the latent embeddings from both unimodal encoders and passes the output to the unimodal decoders. This setup enables the model to learn a shared representation through backpropagation.

### Ablation and hyperparameter analysis

**Fig. 2:**
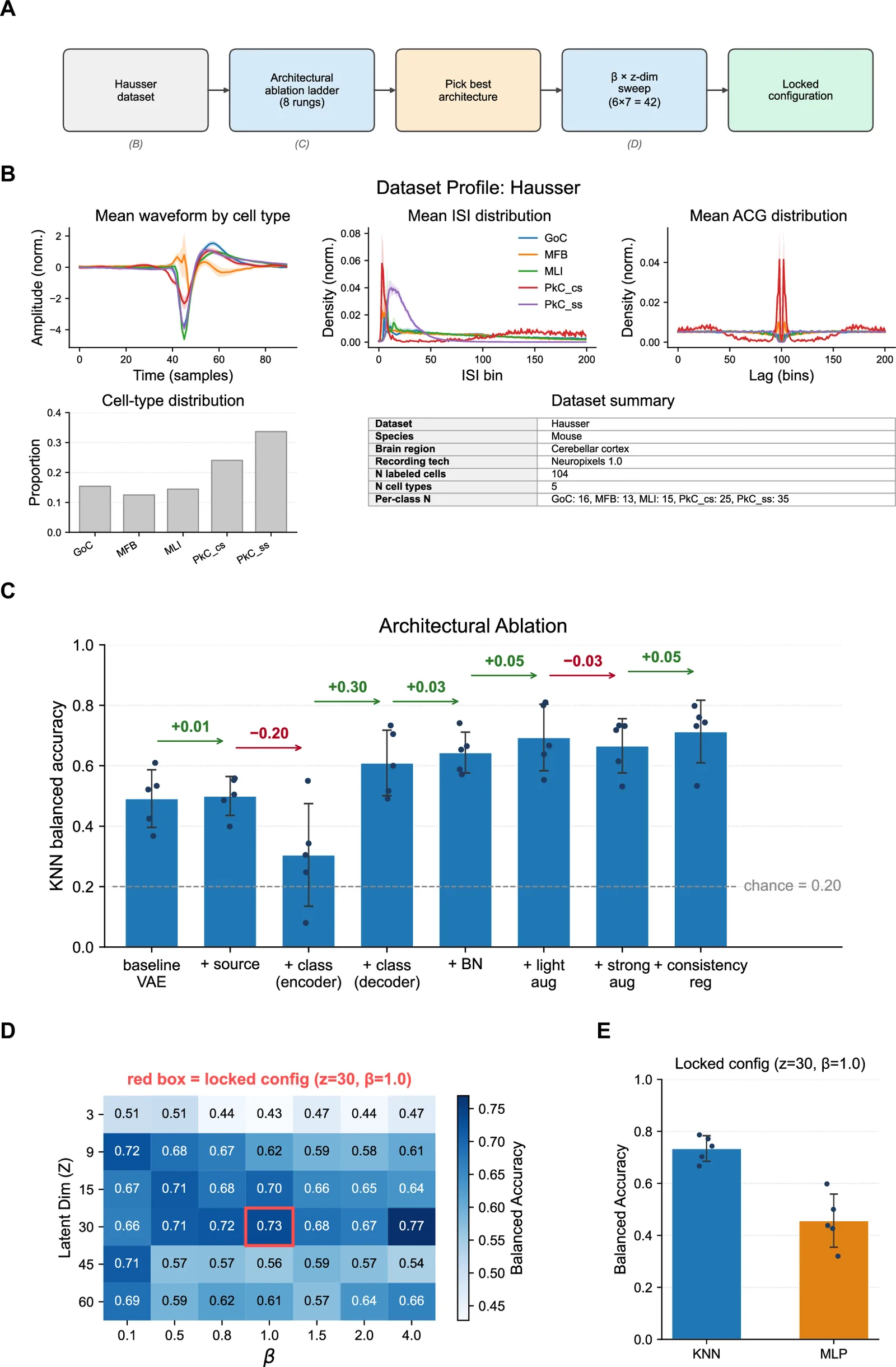
Ablation and hyperparameter tuning. All panels use the Hausser mouse cerebellar cortex dataset^23^: *N* = 104 labeled neurons across 5 cell types. The unit of study is the single spike-sorted neuron, an independent biological unit contributing one entry. Balanced accuracy is estimated by stratified fivefold cross-validation over these 104 neurons, so the replicates underlying every bar-chart error bar are the *n* = 5 folds (a computational resampling of one fixed set of neurons, not 5 independent experiments). **A** Pipeline schematic of the ablation and hyperparameter analysis. **B** Average feature distribution per cell type (mean waveform, ISI distribution and ACG, top), cell type distribution (bottom left) and a summary of the dataset and its per-class counts (bottom right) for the Hausser (mouse cerebellar cortex) dataset. Traces show the per-cell-type mean; shaded bands show ± 1 standard deviation across the neurons of that cell type (*n* = 16 GoC, 13 MFB, 15 MLI, 25 PkC_cs, 35 PkC_ss). **C** Ablation ladder for eight architectural variants evaluated on the Hausser dataset. Bars show the mean balanced accuracy and error bars ± 1 standard deviation (*N* = 104 neurons; chance = 0.2 for 5 classes). **D** Grid plot for the hyperparameter sweep over *β* and latent dimensionality (*z*-dim) on the Hausser dataset, with the locked configuration (*z* = 30, *β* = 1.0, KNN balanced accuracy 0.734) marked. Each cell shows the mean balanced accuracy across the *n* = 5 folds for that configuration, printed in the cell and encoded by the accompanying color bar; the locked configuration is outlined in red. **E** KNN vs. MLP classification head at the locked configuration. Bars show the mean balanced accuracy and error bars ± 1 standard deviation (KNN 0.734 ± 0.049; MLP 0.457 ± 0.102). BN batch normalization, Aug augmentation, reg regularization. *z*-dim latent dimensionality. KNN k-nearest neighbors, MLP multilayer perceptron, GoC Golgi cell, MFB mossy fiber bouton, MLI molecular layer interneuron, PkC_ss Purkinje cell simple spike, PkC_cs Purkinje cell complex spike. Colors denote cell type as given in the (**B**) legend and are used consistently across panels. Source data are provided as a Source Data file.

We conducted ablation studies evaluating architectural components, hyperparameters, and cross-dataset robustness of HIPPIE (Fig. 2A). For these tasks we used the Hausser mouse cerebellar cortex dataset^23^, which contains recordings from 104 labeled neurons across 5 cell types: Golgi cells (GoCs), mossy fibers (MFBs), molecular layer interneurons (MLIs), and Purkinje cells (simple spikes - PkC_ss and complex spikes—PkC_cs) (Fig. 2B; more details in “Methods”).

We evaluated eight architectural variants from the base architecture to a regularized model with multilabel conditioning, isolating the impact of specific design choices (Fig. 2C).

The baseline unconditioned VAE achieved 0.491 balanced accuracy. Adding source conditioning left performance unchanged (+0.009; 0.500), as expected for a single-source dataset where no cross-source normalization is required. We then compared two strategies for introducing class information, each tested from the source-conditioned baseline: encoder-only conditioning reduced performance by −0.195 (0.305 ± 0.170), while decoder-level conditioning improved it by +0.109 (0.609 ± 0.108) (Fig. 2C). Building from the decoder configuration, batch normalization contributed +0.035 (0.644). We then compared two augmentation regimes from that base: light augmentation improved performance by +0.050 (0.694), while strong augmentation provided a smaller gain of +0.022 (0.666); light augmentation was therefore retained. Consistency regularization applied on top of light augmentation added +0.019, reaching a final balanced accuracy of 0.713 ± 0.104. Class conditioning at the decoder, rather than source conditioning or regularization, is the dominant architectural driver of discriminative performance.

We then performed a sweep of 42 hyperparameter configurations spanning latent dimensionality (*z*-dim) and KL-divergence weighting (*β*) (Fig. 2D). Performance was stable across most of the swept region, with higher balanced accuracy concentrated at mid-to-high *z*-dim values and *β* ≈ 1.0. Although the highest mean was achieved at *β* = 4.0 (0.770 ± 0.117), its variance was substantially larger than at *β* = 1.0 (0.734 ± 0.049); the stability tiebreaker selected the more reliable configuration (highest mean within 1.96 SEM of the global best, with lowest standard deviation as the tiebreaker; see “Methods”). The locked configuration (*z* = 30, *β* = 1.0) was frozen before any other benchmark dataset was evaluated.

We compared KNN and MLP classification heads at the locked configuration on the Hausser dataset (Fig. 2E). KNN (balanced accuracy 0.734 ± 0.049) outperformed MLP (0.457 ± 0.102). This gap likely reflects the multimodal local structure of the learned embedding space, where a single biological cell type produces multiple distinct electrophysiological signatures depending on recording geometry or functional state. KNN naturally accommodates this distributed structure through local neighborhood consensus, whereas the MLP’s global decision boundary cannot capture within-class variation, though this may not hold for larger datasets.

Throughout, we compare HIPPIE to previously published methods^21,24^. For a fair comparison, NEMO and PhysMAP underwent matched hyperparameter sweeps on the same dataset. Best configurations based on balanced accuracy and stability were selected: NEMO (*τ* = 0.5, lr = 10^−4^, *z* = 256), PhysMAP (dimV = 5, distance = euclidean, nfeatures = 20) (Supplemental Figs. S1 and S2; “Methods”).

### Cell-type classification from waveform and spike-timing features

To assess HIPPIE’s ability to resolve cell-type-specific electrophysiological signatures, we analyzed three datasets combining in vivo extracellular recordings with optogenetic tagging of defined neuronal subtypes. We also included a dataset with expert-assigned Fast-spiking and Regular-spiking labels. These datasets encompassed distinct recording technologies and brain regions: the Extracellular Mouse A1 Dataset^31^, recorded from primary auditory cortex with a silicon probe (285 neurons, 3 cell types), the Juxtacellular Mouse S1 Cell Type Dataset^32^, recorded with glass micropipettes from the mouse primary somatosensory cortex (224 neurons, 5 cell types), Ramachandran dataset^33^, recorded from rat somatosensory cortex with 32-channel NeuroNexus electrodes (134 neurons, Inhibitory vs Excitatory) and the Calvigioni dataset, recorded from the mouse prefrontal cortex with Neuropixels (9213 neurons, Fast spiking vs Regular spiking).

We compared HIPPIE against established methods for electrophysiological cell type classification across the four datasets. For the Extracellular Mouse A1 and Juxtacellular Mouse S1 Cell Type datasets, we used extracellular waveforms and Interspike Interval distributions. For the Ramachandran and Calvigioni datasets, we used the Interspike Interval distributions and autocorrelograms. Baselines included: principal component analysis on mean waveform (PCA-WF), principal component analysis on inter-spike interval distribution (PCA-ISI), principal component analysis on autocorrelogram (PCA-ACG), PhysMAP^21^, and a bimodal VAE trained on the same features as HIPPIE. As negative controls, we performed random shuffling and shifting of the training labels (see “Methods”).

**Fig. 3:**
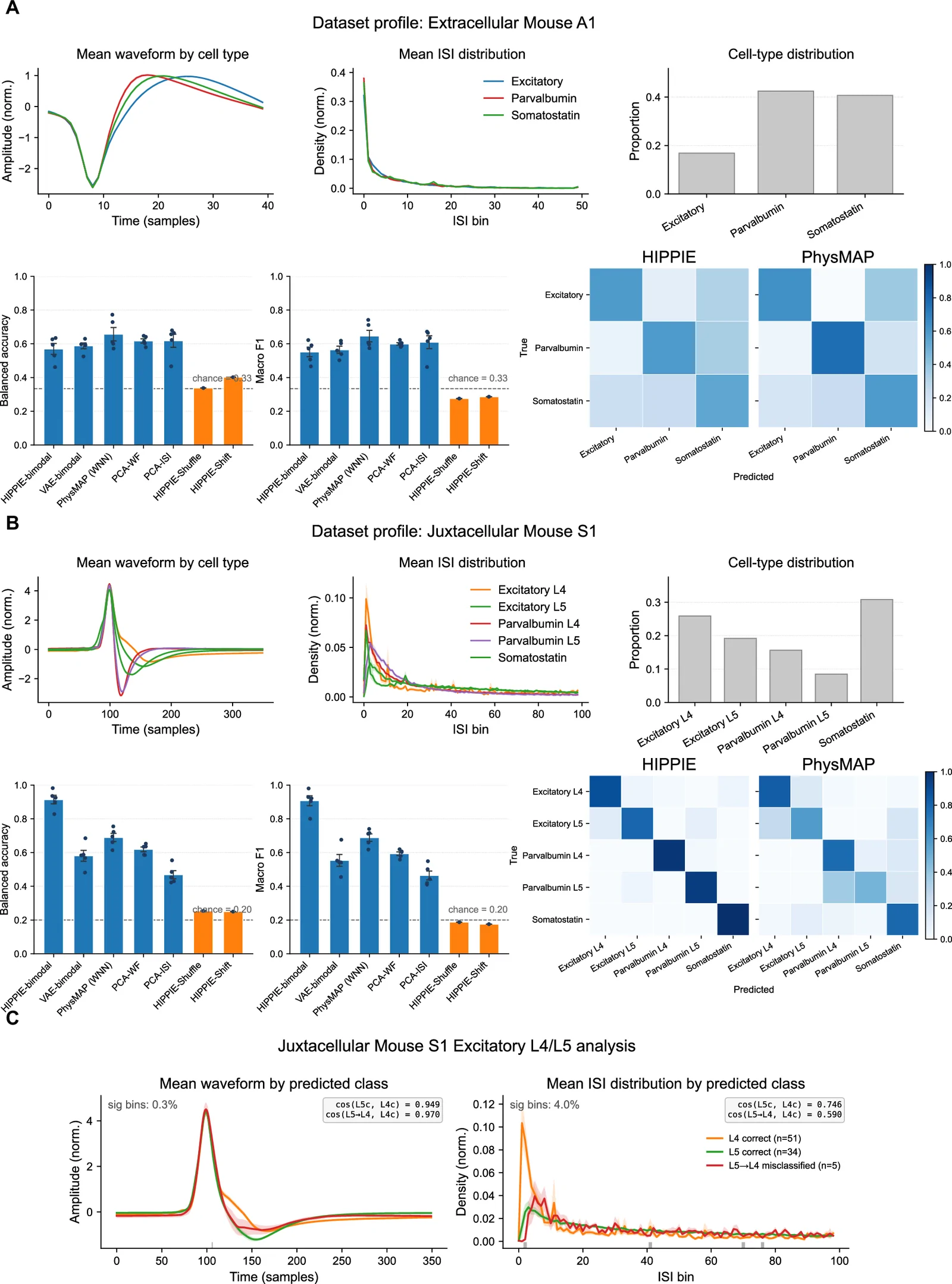
Cell-type classification using waveform and ISI features. In all panels, the unit of study is the single spike-sorted neuron; *N* is a count of neurons, which are independent biological units each contributing one entry. **A** Feature distribution per cell type and cell type distribution for the Extracellular Mouse A1 dataset (*N* = 285 neurons across 3 cell types; Excitatory: 48, Parvalbumin: 121, Somatostatin: 116), and accuracy and Macro F1 benchmark comparison for the same dataset (chance balanced accuracy = 0.333). **B** Feature distribution per cell type and cell type distribution for the Juxtacellular Mouse S1 dataset (*N* = 224 neurons across 5 cell types; Layer 4 Excitatory: 58, Layer 5 Excitatory: 43, Layer 4 Fast-spiking: 35, Layer 5 Fast-spiking: 19, Somatostatin: 69), and accuracy and Macro F1 benchmark comparison for the same dataset (chance balanced accuracy = 0.200). **C** Analysis of Excitatory Layer 4 and Layer 5 neurons and their misclassification. Layer 4 correct: *n* = 51, Layer 5 correct: *n* = 34, Layer 5 to Layer 4 misclassified: *n* = 5, where each *n* counts individual neurons pooled across the 5 cross-validation test folds (every neuron is a test-set member exactly once). In (**A** and **B**), feature traces show the per-cell-type mean ± 1 standard deviation across the neurons of that cell type (biological units; per-class *n* as listed above). The benchmark bar charts in (**A** and **B**) present mean ± 1 standard deviation across the *n* = 5 folds of a stratified fivefold cross-validation; these folds are a computational resampling of one fixed set of neurons and quantify estimate stability rather than independent biological replication. Blue bars are the benchmarked methods and orange bars the two negative controls (HIPPIE-Shuffle, cell-type labels randomly permuted before training; HIPPIE-Shift, each input feature vector circularly shifted by a random offset). Each panel also shows confusion matrices for HIPPIE and PhysMAP, pooled across the 5 cross-validation test folds so that every neuron appears exactly once, row-normalized to the true-class recall, with a shared color scale from 0 to 1. WF mean waveform, ISI interspike interval distribution, PCA principal component analysis, KNN k-nearest neighbors, WNN weighted nearest neighbors, VAE unconditioned variational autoencoder, A1 primary auditory cortex, S1 primary somatosensory cortex. Colors denote cell type as given in each panel legend. Source data are provided as a Source Data file.

On the Extracellular Mouse A1 Dataset^31^, PhysMAP achieved the highest balanced accuracy (0.657), followed by PCA-ISI (0.618), PCA-WF (0.617), VAE-bimodal (0.588), and HIPPIE-bimodal (0.569 ± 0.073) on this three-class task (chance = 0.333) (Fig. 3A). The spread across all methods is narrow (0.088), and even the best method (PhysMAP) sits only 0.324 above chance, reflecting a shared performance ceiling imposed by within-class variability from silicon-probe recordings of three cell types that differ mainly in waveform kinetics and mean firing rate.

For the Juxtacellular Mouse S1 Cell Type Dataset^32^, HIPPIE-bimodal achieved the highest balanced accuracy (0.914 ± 0.057), outperforming PhysMAP (0.689), PCA-WF (0.619), VAE-bimodal (0.581), and PCA-ISI (0.469). All methods exceeded random chance (0.200) (Fig. 3B). The S1 experiment included layer information among HIPPIE’s conditioning variables, so the improved performance reflects the full conditional framework’s ability to integrate electrophysiological features with informative metadata, rather than waveform and spike-timing features alone.

The bimodal benchmarks for Calvigioni and Ramachandran datasets are shown in Supplemental Fig. S3; all methods performed within 0.028 balanced accuracy of each other on Calvigioni, while variance was high across methods on the class-imbalanced Ramachandran dataset.

The Juxtacellular Mouse S1 Cell Type dataset contains optotagged identity (excitatory projection neurons, Pvalb and Sst interneurons) and layer information^32^, providing an opportunity to test whether HIPPIE embeddings capture variation beyond cell type labels. In primary somatosensory cortex, Layer 4 (L4) excitatory neurons are predominantly spiny stellate cells that exhibit regular spiking patterns^34,35^. Layer 5 (L5) contains two principal excitatory subtypes with distinct properties: callosal projection neurons (CPNs) and subcerebral projection neurons (SCPNs). L5 SCPNs are characterized by thick apical dendrites and intrinsic bursting or burst-adapted firing patterns, while CPNs have thinner dendrites and regular spiking patterns^36,37^. We leveraged this known heterogeneity to test whether L5 neurons that HIPPIE classified as L4 represent errors or reflect underlying biological variation. Of 43 L5 excitatory neurons, 34 were correctly classified, 5 were assigned to L4, and 4 to fast-spiking (Fig. 3C). These misclassified neurons had waveforms more similar to the L4 mean than correctly classified L5 neurons (cosine similarity 0.970 vs. 0.949), yet their ISI profiles remained dissimilar to L4 (0.590 vs. 0.746), suggesting that HIPPIE’s classification was driven by spike morphology rather than firing dynamics in this subgroup, consistent with the known electrophysiological diversity of L5 excitatory subtypes rather than simple misclassification.

### HIPPIE generalizes to multiple electrophysiological modalities

To evaluate scalability to three electrophysiological modalities, we extended the cVAE architecture to incorporate autocorrelograms alongside waveforms and ISI distributions, training HIPPIE on all three modalities. We included NEMO^24^, a method employing a CLIP-based contrastive objective to align waveform and ACG representations. NEMO trains separate encoders to embed augmented views from the same neuron close together while separating different neurons, allowing comparison between conditioned generation and contrastive learning.

For the trimodal benchmark, we used three datasets where all three modalities were available: the Hull mouse cerebellar cortex dataset (103 neurons, 5 cell types), the Lisberger macaque cerebellar cortex dataset (668 labeled neurons, 5 cell types), described previously in ref. ^23^ and Watson Rat Neocortex dataset (221 neurons, 2 cell types)^38^.

**Fig. 4:**
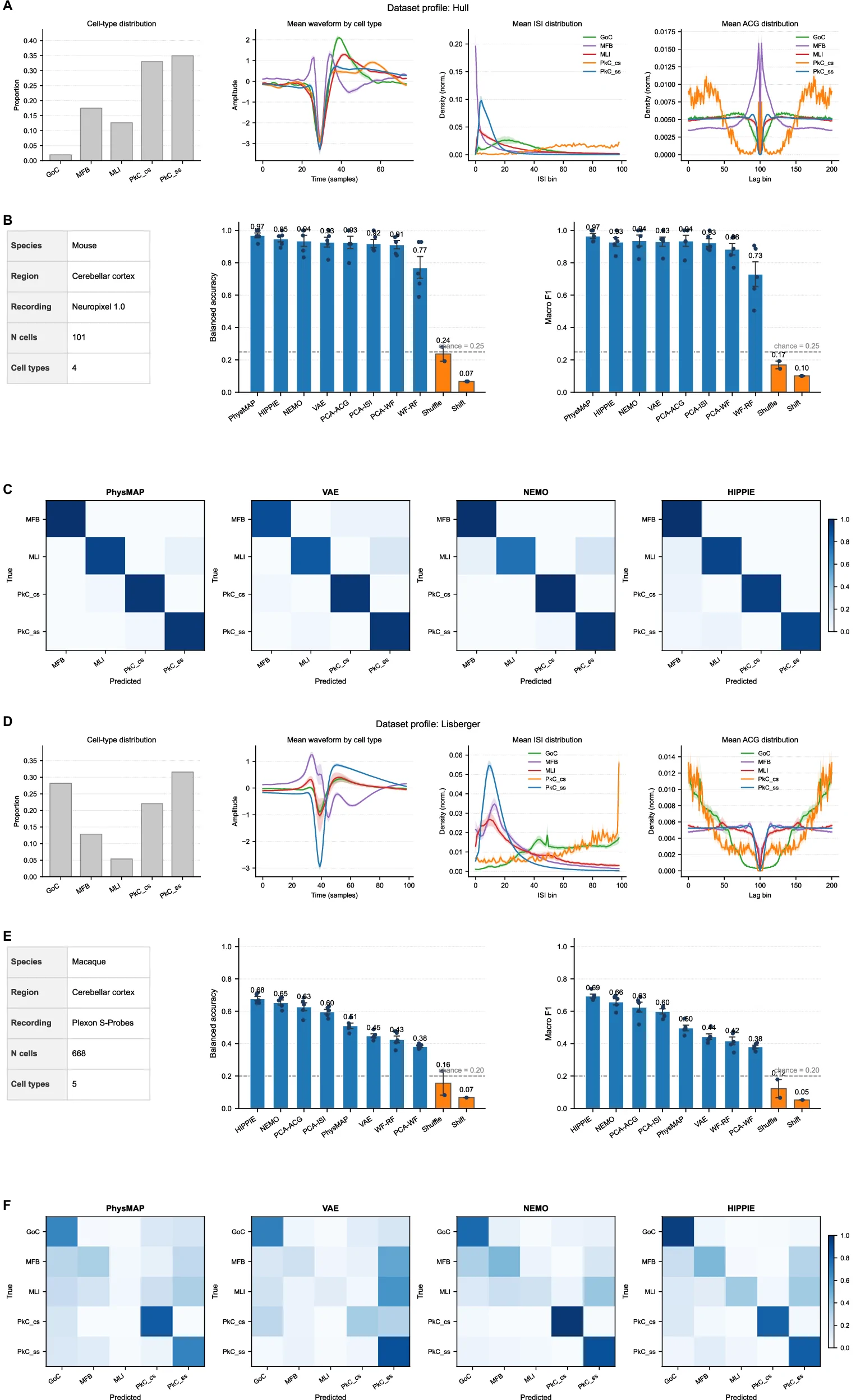
Cell-type classification using waveform, ISI, and ACG features. In all panels, the unit of study is the single spike-sorted neuron; *N* is a count of neurons, which are independent biological units, each contributing one entry. **A** Feature distribution per cell type for the Hull (mouse cerebellar cortex) dataset (*N* = 101 neurons across 4 cell types after removal of rare classes with fewer than 5 neurons; MFB: 18, MLI: 13, PkC_cs: 34, PkC_ss: 36). **B** Balanced accuracy and Macro F1 benchmark comparison for the Hull dataset (*N* = 101 neurons; chance balanced accuracy = 0.25). **C** Confusion matrices for PhysMAP, VAE, NEMO, and HIPPIE on the Hull dataset (*N* = 101 neurons). **D** Feature distribution per cell type for the Lisberger (macaque cerebellar cortex) dataset (*N* = 668 expert-labeled neurons across 5 cell types; GoC: 188, MFB: 86, MLI: 36, PkC_cs: 147, PkC_ss: 211). **E** Balanced accuracy and Macro F1 benchmark comparison for the Lisberger dataset (*N* = 668 neurons; chance balanced accuracy = 0.20). **F** Confusion matrices for PhysMAP, VAE, NEMO, and HIPPIE on the Lisberger dataset (*N* = 668 neurons). In (**A** and **D**), traces show the per-cell-type mean ± 1 standard deviation across the neurons of that cell type (biological units; per-class *n* as listed above), and every labeled class is shown, including classes later removed by the rare-class filter. In (**B** and **E**), data are presented as mean ± 1 standard deviation across the *n* = 5 folds of a stratified fivefold cross-validation; these folds are a computational resampling of one fixed set of neurons and quantify estimate stability rather than independent biological replication. Blue bars are the benchmarked methods and orange bars the negative controls (Shuffle, permuted labels; Shift, randomly circularly shifted inputs). In (**C** and F), confusion matrices are pooled across the 5 cross-validation test folds (each neuron appears in the test set exactly once), row-normalized to the true-class recall, with a shared color scale from 0 to 1. WF mean waveform, ISI interspike interval distribution, ACG autocorrelogram, PCA principal component analysis, WF-RF handcrafted waveform features with a random forest classifier, VAE unconditioned variational autoencoder, GoC Golgi cell, MFB mossy fiber bouton, MLI molecular layer interneuron; PkC_ss Purkinje cell simple spike, PkC_cs Purkinje cell complex spike. Colors denote cell type as given in each panel legend. Source data are provided as a Source Data file.

On the Hull mouse cerebellar cortex dataset (Fig. 4A–C), PhysMAP achieved the highest performance (balanced accuracy 0.969), closely followed by HIPPIE (0.949). NEMO ranked third (0.935), with the baseline VAE close behind (0.927). PCA-based methods all exceeded 0.90: PCA-ACG (0.926), PCA-ISI (0.919), and PCA-WF (0.912). WF-RF (0.770) ranked last among active methods. All methods exceeded chance (0.25) on this four-class dataset after rare (*n* < 5) cell type removal.

On the Lisberger macaque cerebellar cortex dataset (Fig. 4D–F), HIPPIE achieved the highest balanced accuracy (0.679), with NEMO competitive at 0.654. PCA-ACG (0.628) and PCA-ISI (0.598) ranked next, followed by PhysMAP (0.511), the baseline VAE (0.449), WF-RF (0.426), and PCA-WF (0.384). All methods exceeded random chance (0.20). The larger performance spread on the macaque dataset reflects greater within-class variability across 668 neurons and five cell types.

On the Watson dataset (Supplemental Fig. S4), NEMO achieved the highest balanced accuracy (0.81) and macro F1 score (0.82), with HIPPIE competitive at 0.77 balanced accuracy and 0.81 macro F1. PCA-ACG (BA = 0.73) ranked next, followed by PCA-ISI (0.68) and PhysMAP (0.68).

### HIPPIE generalizes across mammalian species via cross-dataset pretraining

**Fig. 5:**
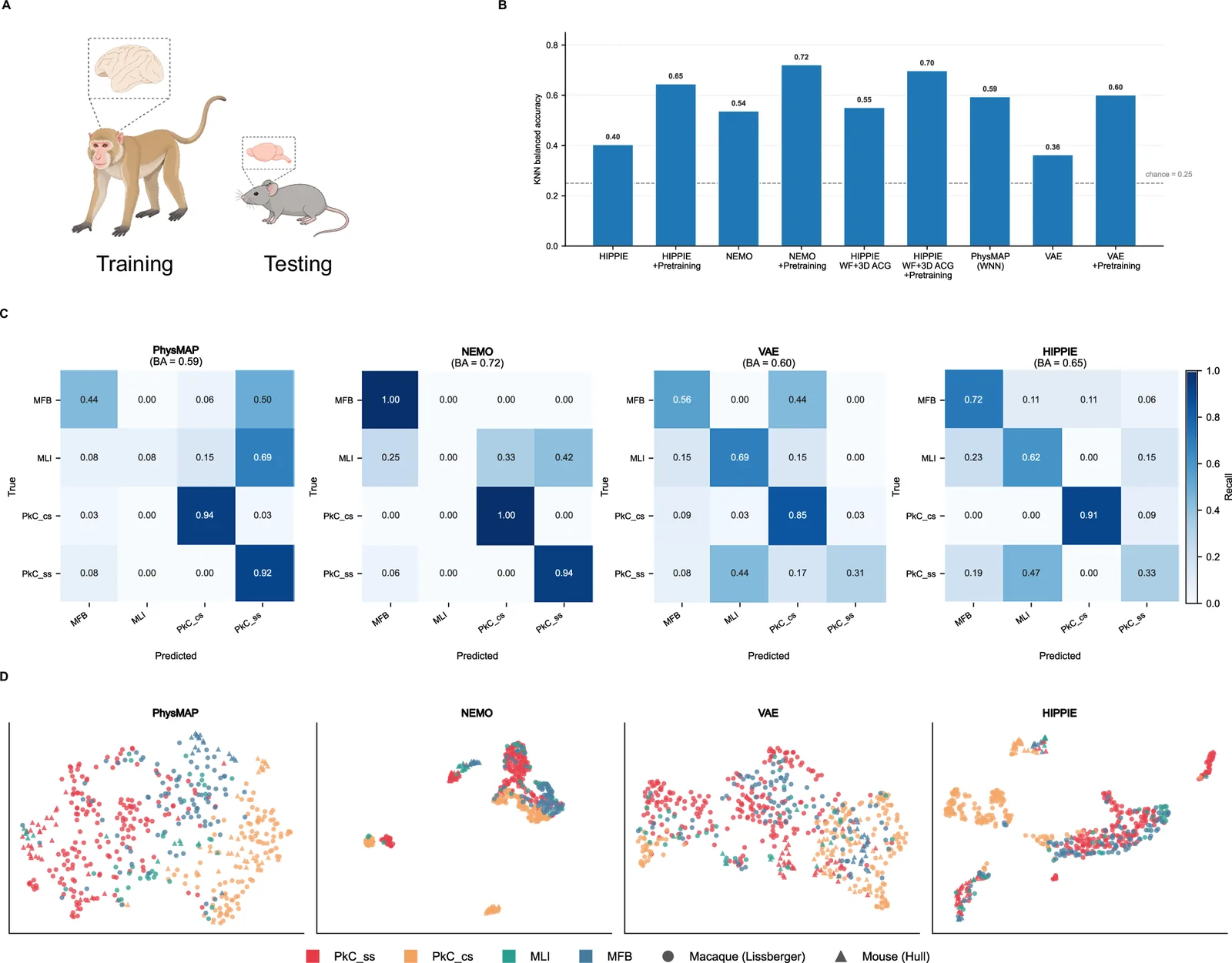
Cross-species generalization via cross-dataset pretraining. All panels show the same cross-species transfer: models trained on the Lisberger macaque dataset and evaluated on the Hull mouse dataset, restricted to the 4 shared cell types. The unit of study is the single spike-sorted neuron; the test set is the complete Hull dataset (*N* = 101 neurons; MFB: 18, MLI: 13, PkC_cs: 34, PkC_ss: 36; chance balanced accuracy = 0.25). Because this is a single macaque-to-mouse transfer rather than a cross-validation, each method yields one value and no error bars are shown. **A** Schematic of the transfer (train on macaque, test on mouse). **B** KNN balanced accuracy for each model configuration. HIPPIE, HIPPIE with a waveform and 3D-autocorrelogram input (WF + 3D ACG), NEMO and the unconditioned VAE are each shown with and without unlabeled pretraining; PhysMAP (WNN) has no pretraining stage and appears once. **C** Confusion matrices for PhysMAP and for the pretrained NEMO, VAE and HIPPIE models, whose balanced accuracies (BA, printed above each matrix) correspond to the matching bars in (**B**). Matrices are row-normalized to the true-class recall, with a shared color scale from 0 to 1. **D** Joint UMAP of the macaque and mouse embeddings produced by each of the same four methods, colored by cell type. Circles denote macaque (Lisberger) neurons and triangles mouse (Hull) neurons. WNN weighted nearest neighbors, KNN k-nearest neighbors, VAE unconditioned variational autoencoder, BA balanced accuracy, MFB mossy fiber bouton, MLI molecular layer interneuron, PkC_ss Purkinje cell simple spike, PkC_cs Purkinje cell complex spike. Source data are provided as a Source Data file.

To evaluate cross-species generalization, we trained models on macaque cerebellar cortex (Lisberger) and tested on mouse recordings (Hull), restricting to four shared cell types (PkC_ss, PkC_cs, MLI, MFB; chance = 0.25) (Fig. 5A). Among methods with cross-dataset pretraining, NEMO reached the highest balanced accuracy (0.72), followed by HIPPIE (0.65) and the baseline VAE (0.60). Cerebellar cell types exhibit distinct temporal firing dynamics^39^. The 3D ACG captures these stereotyped temporal structures, motivating us to adapt HIPPIE to incorporate 3D-ACG features alongside waveforms (HIPPIE 3D + ACG). This raised balanced accuracy to 0.70, closing most of the gap to NEMO (0.72 vs. 0.70). The same featurization advantage held without pretraining, where HIPPIE 3D + ACG (0.55) outperformed both NEMO (0.54) and standard HIPPIE (0.40), though PhysMAP (0.59) led in this regime. Together, these results indicate that featurization is a primary driver of classification performance on this cerebellar dataset, with model architecture playing a secondary role, and that cross-species pretraining is critical for both NEMO and HIPPIE to reach their best performance. Furthermore, because we relied solely on electrophysiological features without auxiliary metadata such as cortical layer information or confidence-based filtering^23^, these findings extend to experimental systems where anatomical context is unavailable, including organoids, 2D cultures, and non-mammalian models.

### HIPPIE’s generative architecture enables cross-species and cross-technology analysis

The cVAE architecture gives HIPPIE capabilities that discriminative models such as NEMO (contrastive encoder, no decoder) and PhysMAP (nonlinear dimensionality reduction, no generative model) cannot provide: signal synthesis, imputation of missing modalities from the remaining inputs, and counterfactual recordings under changed source conditioning. We exploited these properties to ask whether the shared latent space supports generative analysis of cross-species relationships, training a single HIPPIE cVAE jointly on four datasets spanning three species and two brain regions (Hull, Hausser, Watson, Lisberger; no dataset-specific fine-tuning).

**Fig. 6:**
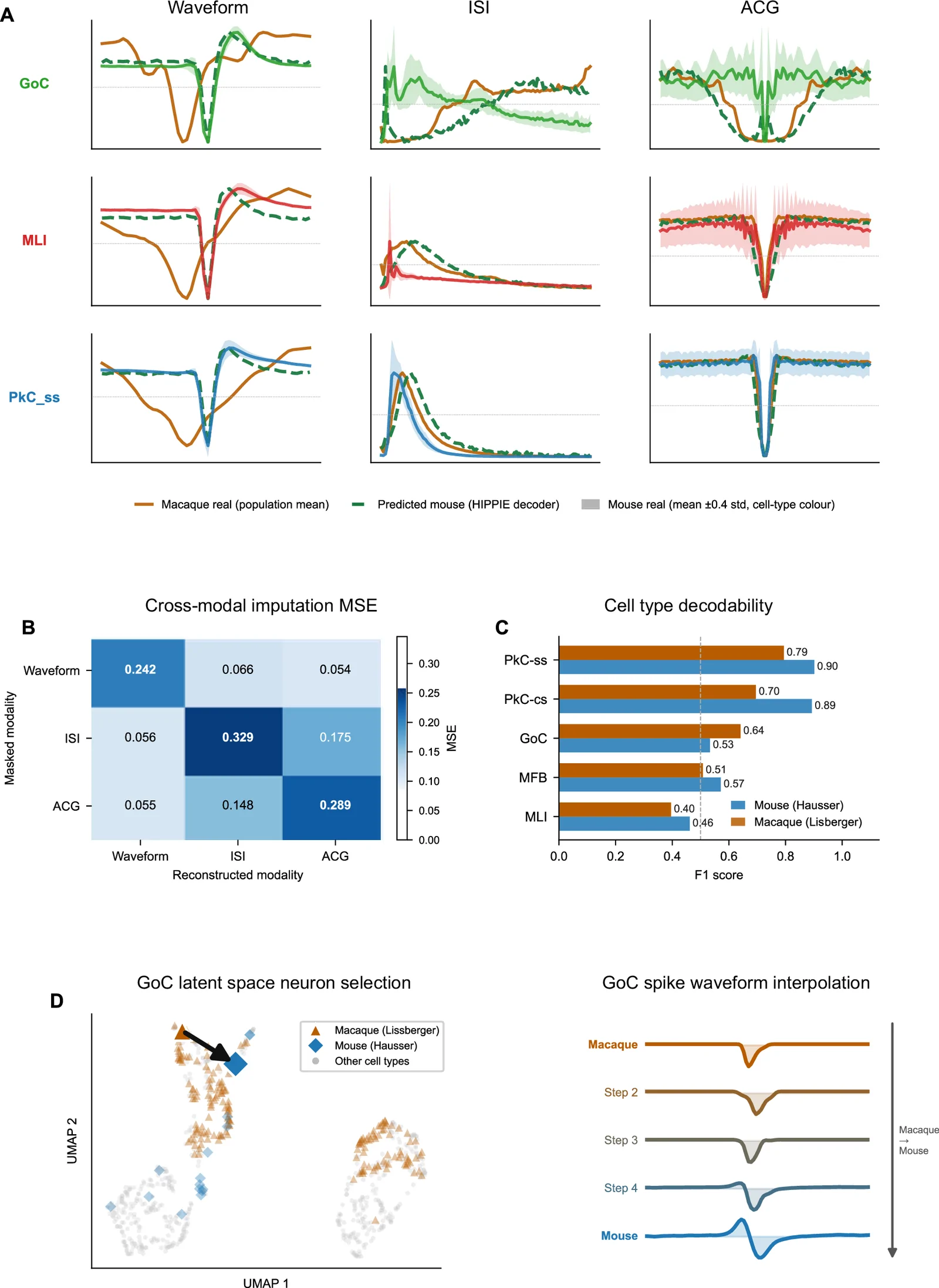
HIPPIE generative capabilities that require a generative decoder. **A** Cross-species counterfactual decoding. Macaque cerebellar neurons (Lisberger, src = 3) were encoded under their true source label and decoded under the mouse cerebellar source label (Hausser, src = 1) for three shared cell types (GoC, MLI, PkC_ss; mouse *n* = 16 GoC, 15 MLI, 35 PkC_ss; macaque *n* = 188 GoC, 36 MLI, 211 PkC_ss) and three modalities. Orange solid = macaque population mean; green dashed = HIPPIE decoder prediction; shaded ribbons = mouse mean ± 0.4 standard deviations across the mouse neurons of that cell type (cell-type color). **B** Cross-modal imputation MSE matrix. Each modality is masked at inference and reconstructed from the remaining two (diagonal); off-diagonal entries show reconstruction of non-masked modalities with one fewer input. Values are the mean squared error, printed in each cell and encoded by the accompanying color bar. **C** Cell-type decodability from the shared latent space. Linear probe F1 scores per cell type for mouse (Hausser, blue) and macaque (Lisberger, orange) from the same checkpoint without fine-tuning. Overall balanced accuracy: 64% (mouse) and 63% (macaque). The dashed vertical line marks an F1 score of 0.5. **D** GoC latent-space geometry. Left: 16-D UMAP of macaque GoC (*n* = 188, orange triangles) and mouse GoC (*n* = 16, blue diamonds), with all other cell types as grey circles for context; the arrow marks the focus pair. Right: decoded waveform waterfall along the straight-line latent interpolation in 5 steps, from the narrow symmetric macaque spike to the broad biphasic mouse GoC waveform. NEMO and PhysMAP have no decoder and cannot perform (**A**–**D**). GoC Golgi cell, MLI molecular layer interneuron, PkC_ss Purkinje cell simple spike, MSE mean squared error, UMAP uniform manifold approximation and projection, SD standard deviation. Source data are provided as a Source Data file.

HIPPIE performs cross-species counterfactual decoding: macaque cerebellar neurons (Lisberger, src = 3) were encoded under their true source label and decoded under the mouse cerebellar source label (Hausser, src = 1) for three shared cell types (GoC, MLI, PkC_ss; Fig. 6A). Across all cell types and modalities, the decoder-predicted mouse signals fall within the real mouse distribution despite the macaque population means being visually distinct. The shared latent space also preserves cross-species geometric structure (Fig. 6D): GoC cells from macaque (*n* = 188) and mouse (*n* = 16) occupy overlapping regions in the 16-dimensional UMAP projection, and decoding along the straight-line interpolation between a focus macaque GoC and its nearest mouse GoC neighbor produces a smooth waveform transition from the narrow symmetric macaque spike to the broad biphasic mouse GoC morphology.

We then used HIPPIE’s joint encoder-decoder as a probe of cross-modal information content. Masking one modality at inference time and asking the model to reconstruct it from the remaining two (Fig. 6B) tests whether the unmasked modalities carry cell-specific information about the masked one. We anchor the test against a population-mean baseline (Supplemental Fig. S6): predicting the per-bin global mean of the masked modality uses only its marginal distribution and no cell-specific information, so beating the baseline indicates that the unmasked modalities contain cell-specific structure about the masked one, while matching or underperforming the baseline indicates that the masked modality’s cell-specific structure is informationally independent of the others.

The analysis reveals an asymmetric information structure across modalities. HIPPIE recovers the ACG from waveform and ISI at lower MSE than the population-mean baseline (0.289 vs. 0.354), indicating that the other two modalities jointly carry cell-specific information about spike-timing autocorrelation. This is consistent with the known relationship between ISI and ACG in renewal-like spike trains^40^, where the ACG at lag *τ* inherits structure from the ISI distribution; accordingly, ISI and ACG partially reconstruct each other from a single modality (off-diagonal MSE 0.148 and 0.175), both beating the mean baseline. By contrast, waveform morphology is largely informationally independent of the spike-timing modalities: HIPPIE does not improve on the mean baseline for ISI or waveform reconstruction, and masking any one modality has little effect on reconstruction of the others across the waveform row and column (off-diagonals 0.054 to 0.066). This result has a direct practical implication: ISI and ACG capture overlapping temporal-firing information and each provides diminishing returns when the other is available, whereas waveform contributes a morphological signal that is not recoverable from spike timing alone. Multimodal integration of all three modalities is therefore supported by information complementarity rather than redundancy.

Cell-type identity remains decodable from the shared latent space without fine-tuning (Fig. 6C): a linear probe achieves balanced accuracy of 64% (mouse) and 63% (macaque) from a single shared checkpoint, with consistent per-cell-type F1 scores across species.

Cross-technology interpolations between A1, S1, and CellExplorer cortical datasets (Supplemental Figs. S5–S8) show smooth waveform transitions across all three recording technologies and cell types; notably, the decoder reproduces dataset-specific ISI window differences when decoding under the CellExplorer source label, confirming that source conditioning captures methodological as well as biological variation. The HIPPIE latent space, therefore, encodes a structured representation of neuronal identity that generalizes across species and recording technologies, supporting generative analysis not available to discriminative models.

### Brain region classification from single-unit features remains challenging across methods

**Fig. 7:**
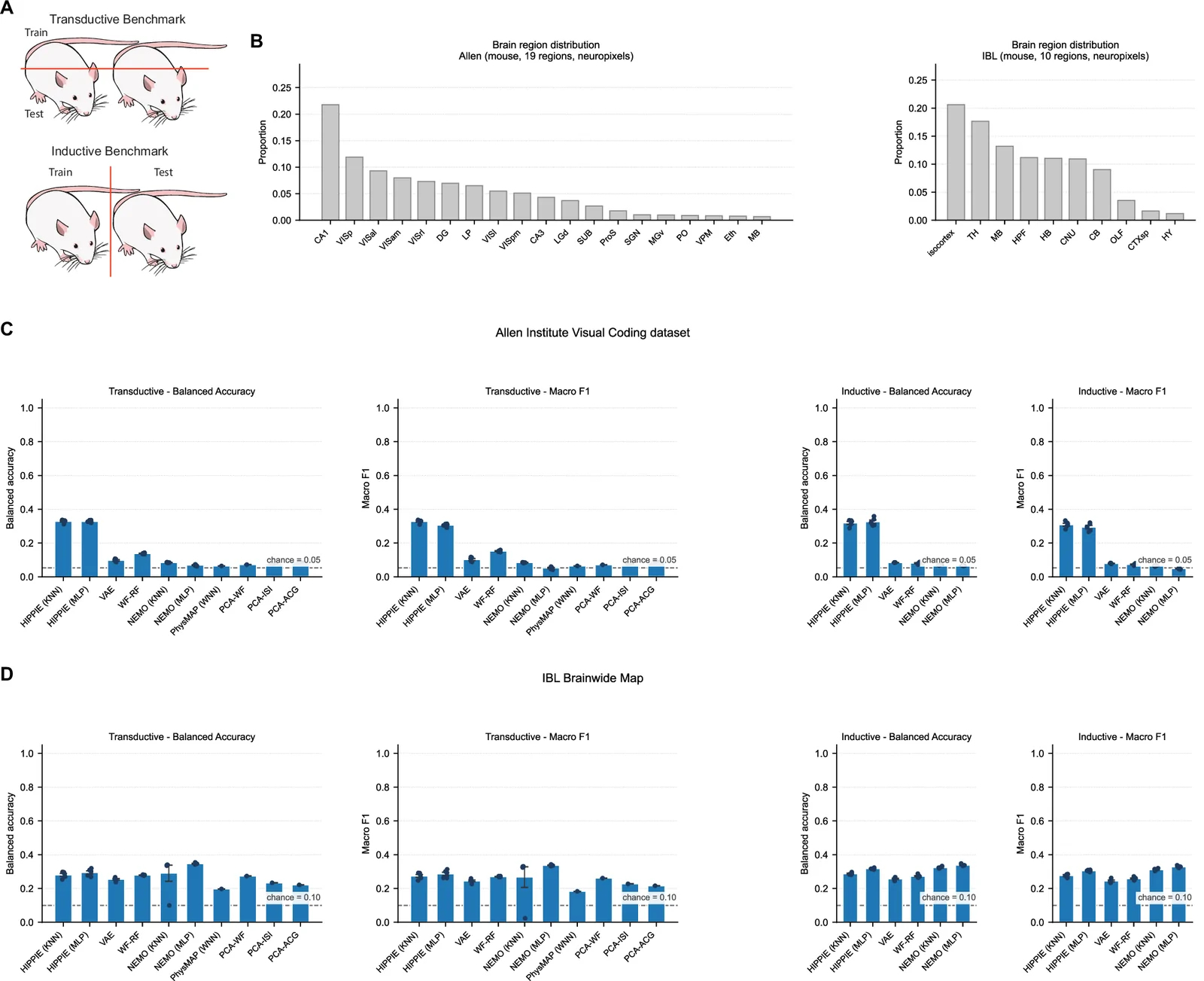
Brain region classification using electrophysiological features. In all panels, the unit of study is the single spike-sorted unit (neuron); *N* is a count of units, which are independent biological observations each contributing one entry. Two datasets are used: the Allen Institute Visual Coding dataset (*N* = 61,781 units from 47 mice, 19 fine-grained regions, chance balanced accuracy = 0.053) and the IBL Brainwide Map (*N* = 62,993 units from 139 subjects and 697 probe insertions, 10 Cosmos regions, chance balanced accuracy = 0.100). **A** Diagram illustrating the difference between transductive and inductive (animal-holdout) evaluation splits. The mouse illustration is “Lab Mouse” (BIOART-000283) by NIAID Visual & Medical Arts, NIAID NIH BIOART Source (https://bioart.niaid.nih.gov/bioart/283). **B** Proportion of units per brain region in the Allen Visual Coding dataset (left; 19 fine-grained regions) and in the IBL Brainwide Map (right; 10 Cosmos regions). **C** Balanced accuracy and Macro F1 scores per method using KNN and MLP probes on the Allen Institute Visual Coding dataset, in the transductive and inductive settings. **D** The same four comparisons on the IBL Brainwide Map. For the Allen evaluation HIPPIE was conditioned on the known broad super-region during both training and inference, whereas the IBL evaluation used no region conditioning. In (**C** and **D**), bars show the mean and error bars ± 1 standard deviation across the *n* = 5 folds of a fivefold cross-validation; overlaid points give the individual fold values. Transductive folds are stratified over units; inductive folds are grouped by animal, so every test unit comes from an animal absent from that fold’s training set, and each unit is held out exactly once. The held-out animals are biologically independent of the training animals, and the fivefolds are a computational resampling, not 5 independent experiments. PCA-based methods and PhysMAP appear only in the transductive setting, as they fit projections directly to the data and cannot embed unseen animals. KNN k-nearest neighbors, PCA principal component analysis, WF-RF handcrafted waveform features with a random forest classifier, VAE unconditioned variational autoencoder, IBL International Brain Laboratory. Source data are provided as a Source Data file.

Brain region prediction from single-unit electrophysiological features is an open challenge, as neurons in different regions can share similar intrinsic properties while location-specific signatures may be distributed across populations rather than individual cells. We evaluated this task on two large-scale datasets: the Allen Institute Visual Coding dataset^41^ (61,781 units, 47 mice, 19 brain regions) and the IBL Brainwide Map (62,993 neurons, 139 subjects, 10 Cosmos regions) (Fig. 7A, B), using all three modalities. We employed transductive (80–20 stratified by brain area) and inductive (animal-holdout; 37 training mice, 10 held-out animals) evaluation paradigms. PCA-based methods and PhysMAP were restricted to the transductive setting, as they fit projections directly to the available data and cannot embed recordings from unseen animals. For the Allen dataset, HIPPIE was conditioned on the known broad super-region (a five-way grouping of the 19 fine areas: visual cortex, hippocampus, thalamus, midbrain, and other) during both training and inference, as experimenters typically have access to this coarse anatomical context from probe targeting; the IBL Cosmos-level evaluation used no region conditioning.

On the Allen dataset (19 fine-grained regions), HIPPIE achieved balanced accuracy 0.319 ± 0.009 (inductive) and 0.329 ± 0.004 (transductive), outperforming all inductive-eligible baselines: PhysMAP 0.163 and NEMO 0.071, the latter near random chance (0.053) (Fig. 7C, D). On the IBL dataset (10 Cosmos regions), NEMO led in the inductive setting (0.324 ± 0.003), followed by HIPPIE (0.287 ± 0.004), WF-RF (0.272), and VAE (0.257); all exceeded random chance (0.100). The stronger performance of PCA methods in the transductive IBL setting relative to Allen may reflect that 10 broad Cosmos regions are more separable by simple feature statistics than 19 fine-grained Allen areas, though this remains to be tested systematically. These results suggest that on the Allen dataset, broad super-region conditioning provides a meaningful signal for anatomical localization, while HIPPIE remained competitive with the best baseline on the unconditioned IBL task; fine-grained brain region prediction may ultimately require population-level features such as local field potentials or inter-unit correlations.

### A web application for HIPPIE inference and analysis

**Fig. 8:**
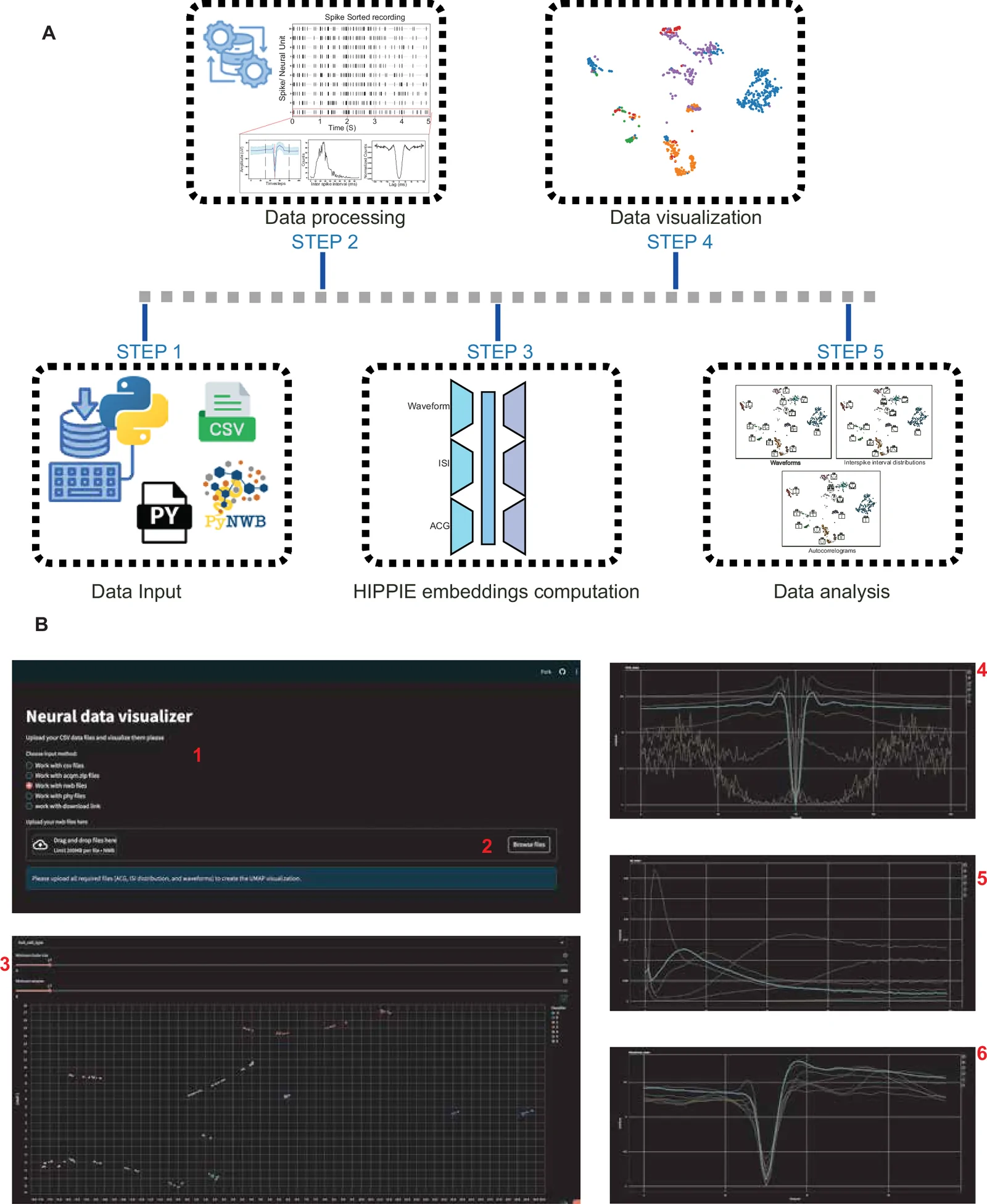
The HIPPIE web application for inference and clustering. **A** Diagram illustrating the web application workflow. Data processing icon was obtained from Flaticon.com. **B** Interface panels showing (1) input format selection, (2) file upload, (3) UMAP visualization with HDBSCAN parameter controls, and (4–6) cluster-averaged ACG, ISI distribution, and waveform. Panels are screenshots of the application interface rather than plotted results, so no source data file accompanies this figure. UMAP uniform manifold approximation and projection, HDBSCAN hierarchical density-based spatial clustering of applications with noise, ISI interspike interval distribution, NWB (Neurodata Without Borders).

Many neuroscience laboratories lack the computational expertise to deploy deep learning models, which limits the practical reach of methods like HIPPIE; we therefore developed an open web application for HIPPIE inference and clustering, available at https://hippie-web.streamlit.app/. Users upload recordings in standard formats (NWB, PHY/Kilosort output zip, CSV, or ACQM) and obtain embeddings from a pretrained model trained on all datasets presented in this study. A recording-technology selector (Neuropixels 1.0/2.0, silicon probe, juxtacellular) conditions the encoder on the recording hardware, matching the three-class vocabulary used during pretraining. The application supports unsupervised clustering, interactive UMAP visualization, and inspection of cluster-averaged waveforms, autocorrelograms, and ISI distributions (Fig. 8). Datasets up to 2 GB are supported, accommodating typical multi-electrode array experiments. The Python package and web application together make HIPPIE accessible to experimentalists regardless of their computational background.

## Discussion

HIPPIE’s conditioning on recording technology, species, and brain region separates biological signal from technical noise in a framework that supports both classification and generative analysis, enabled by its conditional generative decoder. On the cross-species benchmark, NEMO with pretraining achieved the highest balanced accuracy, followed by HIPPIE; without pretraining, both methods dropped substantially, confirming that multi-species pretraining is critical for transfer. HIPPIE’s advantage over NEMO is not in classification rank but in its decoder: the source-conditioning mechanism synthesizes what a macaque cerebellar neuron would look like if recorded in the mouse and places GoC, MLI, and PkC_ss cells from both species in a shared latent neighborhood; its contribution is interpretable generative transfer, not raw discriminative performance. That cell types conserved across ~80 million years of evolutionary divergence remain separable in a shared latent space suggests intrinsic electrophysiological properties reflect conserved biophysical constraints. Electrode placement relative to the soma can alter waveform polarity and shape, and available datasets do not yet span the recording geometries seen in practice; our polarity analysis (Supplementary Tables S1 and S2) found no systematic relationship between polarity composition and classification accuracy across or within datasets, suggesting the source-conditioning layer absorbs recording-geometry effects rather than allowing them to confound biological classification, though this needs testing as more diverse datasets become available.

Reproducibility in electrophysiology extends beyond cross-species transfer to within-species consistency across laboratories and experimental conditions^42^. The cross-technology latent-space interpolations between A1, S1, and CellExplorer cortical recordings (Supplemental Figs. S5–S8) show the source-conditioning layer captures methodological differences across recording systems, yielding consistent embeddings across laboratories with different pipelines. The cross-modal analysis shows that ISI and ACG share information through their common origin in the spike train, while waveform morphology is largely independent of both; HIPPIE recovers the autocorrelogram from the other two modalities but does not improve on a population-mean baseline for ISI or waveform reconstruction. This asymmetry is a finding about the information geometry of extracellular electrophysiology rather than a limitation of the model, and it justifies multimodal integration: the three modalities span two largely non-overlapping information dimensions, so none can substitute for the others.

To lower the barrier to adoption, we provide a Streamlit-based web application for rapid clustering and neuronal identity inference without programming expertise^43,44^. Applying HIPPIE to new data requires no hyperparameter tuning: the fixed defaults (*β* = 1.0, *z* = 30) selected on the Hausser dataset held across all 11 benchmark datasets without per-dataset adjustment. Laboratories working with species or brain regions not covered by current training data can fine-tune from the pretrained checkpoint at https://huggingface.co/Jesusgf23/hippie using the same fixed defaults as an initialization. Projects focused on generation can use the generative defaults (*β* = 0.1, *z* = 16).

Beyond basic neuroscience, electrophysiological classification tools have direct neurotech applications. In brain-computer interfaces, identifying the brain region and cell types generating signals improves interpretation of user intent^45^. HIPPIE’s cross-species performance suggests it could classify human recordings from animal-model training data, enabling real-time cell-type identification during clinical recordings.

The present study has three limitations. First, HIPPIE’s generative capabilities are characterized here rather than deployed: counterfactual decoding, cross-species interpolation, and cross-modal imputation behave as expected, but their value on a concrete downstream task remains untested. Second, labeled human neuronal data are absent from training, so HIPPIE’s potential for clinical applications rests on the assumption of cross-species transferability, which our experiments support but do not guarantee at the human scale. Third, the moderate absolute accuracy on brain region classification (Allen inductive: 0.319, IBL inductive: 0.287) likely reflects the inherent ambiguity of single-unit features for fine-grained anatomical localization; population-level signals such as local field potentials or inter-unit correlations are a natural next step. More broadly, which featurizations of extracellular neural activity are most informative for cell-type and brain-region classification remains an open question, since mean waveform shape, ISI distribution, and ACG have been adopted largely by convention; expanding coverage across brain areas, recording technologies, and species beyond the three represented here will be necessary before the framework can be considered broadly representative of the field, and we view this as a community effort that the shared benchmarking repository is built to support.

## Methods

### Data processing pipeline

Per-unit features were extracted from each dataset through source-specific pipelines. For the C4 database (Hausser, Hull, and Lisberger datasets), H5 files were parsed with custom scripts to extract per-neuron mean waveforms, spike trains, and cell-type labels. For the Allen Institute Visual Coding dataset, sessions were downloaded via AllenSDK and mean waveforms were extracted from the pre-computed per-unit representations. For the DANDI Archive datasets (Watson, Ramachandran, and Calvigioni), waveforms and spike trains were extracted directly from NWB files; scripts are available at https://github.com/braingeneers/HIPPIE and serve as a starting point for any NWB-formatted dataset. For the IBL Brainwide Map, quality-filtered units were retrieved via the ONE API. Raw recordings requiring spike sorting from scratch can be processed using the pipeline described in ref. ^27^.

For all sources, waveforms were extracted from the peak channel (maximum peak-to-trough amplitude), trough-centered with the trough at one-third of the window, and normalized to [−1, 1]. All modalities are resampled at load time to a common fixed size via linear interpolation (torch.nn.functional.interpolate, mode = linear): waveform to 50 samples (2.5 ms at an effective 20 kHz), ISI distribution to 100 bins (0–100 ms window), and ACG to 100 bins (±100 ms window, resampled from 201 extracted bins at 1 ms resolution). Waveform averaging used up to 500 raw spikes per unit. ISI distributions are additionally log-transformed (log(x+1)) before min-max normalization to [−1, 1].

### HIPPIE’s loss function

The cVAE loss function combines a reconstruction term and a regularization term. The reconstruction loss is the mean squared error (L2) between the input **x** and its reconstruction $\hat{{{\bf{x}}}}$. The Kullback–Leibler (KL) divergence regularizes the latent space by encouraging the encoder distribution to match a standard normal prior. This combination is formalized as:

1 $${{{\mathcal{L}}}}_{{{\rm{VAE}}}}={{\mathbb{E}}}_{{q}_{\phi }({{\bf{z}}}| {{\bf{x}}})}[\log {p}_{\theta }({{\bf{x}}}| {{\bf{z}}})]+\beta {D}_{{{\rm{KL}}}}({q}_{\phi }({{\bf{z}}}| {{\bf{x}}})\,| | \,p({{\bf{z}}}))$$

where *q*_*ϕ*_(**z**∣**x**) represents the encoder network (also called the recognition model), *p*_*θ*_(**x**∣**z**) represents the decoder network (the generative model), and *p*(**z**) is the prior distribution.

The hyperparameter *β* controls the trade-off between reconstruction quality and latent space regularity, with *β* = 1 recovering the standard VAE formulation.

When using a Gaussian encoder with diagonal covariance matrix, which is the most common choice, the KL divergence term has a convenient analytical form:

2 $${D}_{{{\rm{KL}}}}({q}_{\phi }({{\bf{z}}}| {{\bf{x}}})\,| | \,p({{\bf{z}}}))=\frac{1}{2}\sum _{j=1}^{J}({\sigma }_{j}^{2}+{\mu }_{j}^{2}-\log ({\sigma }_{j}^{2})-1)$$

where *μ*_*j*_ and *σ*_*j*_ are the predicted mean and standard deviation for the *j*th dimension of the latent space.

Higher values of *β* weight the regularization term more heavily, favoring a more structured latent space at the cost of reconstruction fidelity; lower values prioritize reconstruction quality. HIPPIE conditions the generative process by concatenating learned embeddings for cell-type class, recording technology, and (for the Allen brain-region experiment) super-region to the latent vector **z** before decoding; at evaluation time, these embeddings are masked to zero so the KNN classifier operates on representations that contain no label information. In addition to the ELBO, the production HIPPIE configuration includes a consistency-regularization term: a second decoder pass with the class embedding zeroed produces ${\hat{{{\bf{x}}}}}^{{{\rm{no}}}-{{\rm{class}}}}$, and the loss adds ${\lambda }_{c}{\sum }_{m}{{\rm{MSE}}}({\hat{{{\bf{x}}}}}_{m},{\hat{{{\bf{x}}}}}_{m}^{\,\,{\rm{no-class}}})$ with *λ*_*c*_ = 0.15, scaled linearly over the first 5 epochs. This regularizes the decoder against over-reliance on the class embedding for reconstruction.

### Classification algorithms

KNN classification assigns labels based on majority vote among the *k* training samples closest to each test sample in embedding space. For HIPPIE, embeddings are L2-normalized before classification, and cosine distance is used as the similarity metric. *k* is selected by sweeping *k* ∈ {1, …, 20} and retaining the value that maximizes balanced accuracy on the training fold, evaluated independently per cross-validation iteration. The same per-fold selection is applied to all baselines to keep k-selection rules identical across methods. The PhysMAP implementation realizes the equivalent rule using caret’s internal fivefold structure, picking a fold-local best *k* for each fold. Confidence scores for inference (web application) are derived as the proportion of neighbors sharing the majority class label, ranging from 0 to 1, where higher scores indicate stronger consensus.

MLP probing evaluates embedding quality by training a supervised MLP classifier on the latent representations. Embeddings are standardized with a StandardScaler (fit on the training split) before being passed to an MLPClassifier with hidden layer sizes (256, 128), a maximum of 500 iterations, and early stopping monitoring a held-out 10% validation fraction. While KNN probing assesses the local structure of the embedding space through nearest-neighbor classification, MLP probing tests whether embeddings support structured non-linear decision boundaries.

### Evaluation metrics

We employed KNN and MLP classifiers to evaluate neuronal cell type and brain region classification performance. Balanced accuracy was the primary reported metric; macro F1 was reported as a secondary metric.

#### Balanced accuracy

To account for class imbalance in datasets with unequal representation of cell types, we computed balanced accuracy, which averages the recall across all classes:

3 $${{\rm{Balanced}}}\,{{\rm{Accuracy}}}=\frac{1}{n}\sum _{i=1}^{n}{{\rm{Recall}}}_{i}$$

Where *n* is the number of classes and Recall for each class i is:

4 $${{\rm{Recall}}}_{i}=\frac{{{\rm{True}} \, {\rm{Positives}}}_{i}}{{{\rm{True}} \, {\rm{Positives}}}_{i}+{{\rm{False}} \, {\rm{Negatives}}}_{i}}$$

Balanced accuracy provides equal weight to each class regardless of its frequency in the dataset, making it appropriate for evaluating performance on imbalanced electrophysiological datasets where rare labels may be underrepresented.

#### Macro F1 score

The macro-averaged F1 score provides a comprehensive evaluation across all cell types:

5 $${{\rm{Macro}}}\,{{\rm{F1}}}\,=\frac{1}{n}\sum _{i=1}^{n}F{1}_{i}$$

Where the F1 score for each class is:

6 $$F{1}_{i}=2\cdot \frac{{{\rm{Precision}}}_{i}\cdot {{\rm{Recall}}}_{i}}{{{\rm{Precision}}}_{i}+{{\rm{Recall}}}_{i}}$$

With Precision and Recall defined as:

7 $${{\rm{Precision}}}_{i}=\frac{{{\rm{True}} \, {\rm{Positives}}}_{i}}{{{\rm{True}} \, {\rm{Positives}}}_{i}+{{\rm{False}} \, {\rm{Positives}}}_{i}}$$

8 $${{\rm{Recall}}}_{i}=\frac{{{\rm{True}} \, {\rm{Positives}}}_{i}}{{{\rm{True}} \, {\rm{Positives}}}_{i}+{{\rm{False}} \, {\rm{Negatives}}}_{i}}$$

Performance metrics were estimated using stratified fivefold cross-validation.

### Waveform polarity effect analysis

To assess whether waveform polarity confounds classification accuracy, we classified each neuron’s waveform as negative-going, positive-going, or biphasic using the peak-to-trough amplitude ratio (ratio > 2: positive; < 0.5: negative; otherwise biphasic), computed Pearson correlations between polarity composition and balanced accuracy across datasets, and computed polarity-stratified accuracy within four benchmarks with complete cached predictions (Hull, CellExplorer, Hausser, Lisberger). Between-dataset correlations were not significant for any polarity class (*n* = 9 datasets): % negative, *r*(7) = −0.21, *p* = 0.579, *R*^2^ = 0.046, 95% CI = [−0.77, +0.52]; % biphasic, *r*(7) = −0.06, *p* = 0.885, *R*^2^ = 0.003, 95% CI = [−0.69, +0.63]; % positive, *r*(7) = +0.45, *p* = 0.225, *R*^2^ = 0.202, 95% CI = [−0.31, +0.86], and within-dataset accuracy gaps were inconsistent in direction across datasets, arguing against a systematic polarity artifact (Supplementary Tables S1 and S2).

### Code library details

The HIPPIE pipeline was built using PyTorch Lightning for modularity and reproducibility. Training metrics (accuracy, F1 score, loss) were tracked using Weights and Biases. Two codebases are provided. The user-facing codebase (https://github.com/braingeneers/HIPPIE) supports model training and inference; it includes argparse configuration defaults aligned with the hyperparameter values reported in this “Methods” section (*β* = 1.0, *z* = 30) and masks class-label embeddings to zero at evaluation time to ensure label information is not accessible to the encoder during inference. The benchmarking codebase (https://github.com/JesusGF1/hippie_benchmarking_release) implements fivefold stratified cross-validation and a unified evaluation script that processes HIPPIE and all baselines with identical preprocessing, stratified splits, and KNN evaluation head for PhysMAP, NEMO and HIPPIE, and MLP for NEMO and HIPPIE.

#### HIPPIE training details

For the ablation study and cell-type benchmarks (Figs. 2–4), HIPPIE was trained from scratch on each target dataset using fivefold stratified cross-validation (80% train/20% test); no pretraining from other datasets was performed. For the architecture ablation and hyperparameter tuning experiments, we swept the hidden dimension parameter and *β*, which controls the weight between KL divergence of the latent space against a standard normal distribution and the *L*2 reconstruction error. Fixed defaults of *β* = 1.0 and latent dimensionality *z* = 30 were selected exclusively on the Hausser dataset (42 configurations swept; stability tiebreaker; see below) and frozen before any other benchmark dataset was evaluated, providing outer-loop separation between hyperparameter selection and final performance reporting. Recording technology covariates were included during training. During training, the decoder receives class labels and learns class-specific reconstruction; this forces gradients through the encoder that organize the latent space along class-relevant axes; at inference, the encoder has internalized this structure even without label access, so the KNN classifier operates on representations containing no label information. Each fold produces an independent set of test-set embeddings; reported means and standard deviations are computed across folds. Early stopping monitors reconstruction loss on the held-out fold rather than downstream classification accuracy, preventing decoder overfitting without leaking the evaluation metric into model selection. Training used a learning rate of 10^−3^, up to 100 epochs, batch size 128. The test dataset was loaded only after all training phases completed, and embeddings were extracted using the frozen encoder. The baseline VAE was trained following the same procedure.

For the cross-species transfer experiment (Fig. 5), Lisberger (macaque) served as the source and Hull (mouse) as the target, with evaluation restricted to four shared cell types (PkC_ss, PkC_cs, MLI, MFB; chance = 0.25). Two conditions were compared: in the base condition, HIPPIE was pretrained on Hausser (the remaining C4 dataset, excluding both Lisberger and Hull), fine-tuned on Lisberger, and transferred directly to Hull; in the pretraining variant, Hull was added to the unsupervised pretraining corpus alongside Hausser, giving the model exposure to the target domain before the same Lisberger fine-tuning step. Hull labels were never used during training in either condition.

#### Generative evaluation setup and decoding procedures

For the generative evaluation (Fig. 6), a separate HIPPIE cVAE was trained jointly on four datasets spanning three species and two brain regions: Hull (mouse cerebellar cortex; source_id = 0), Hausser (mouse cerebellar cortex; source_id = 1), Watson (rat frontal cortex; source_id = 2), and Lisberger (macaque cerebellar cortex; source_id = 3). No dataset-specific fine-tuning was applied. Training used a latent dimensionality of *z* = 16, *β* = 0.1 with batch size 256, learning rate 10^−3^, and weight decay 10^−4^. Class-label embeddings were disabled throughout; source conditioning was active and a free-bits constraint of 0.5 nats per latent dimension was applied to prevent posterior collapse.

These hyperparameters differ from the benchmark setting (*z* = 30, *β* = 1.0) because generative and discriminative evaluation have distinct objectives. The reduced dimensionality (*z* = 16) yields a more continuously structured latent space suitable for interpolation and counterfactual decoding, whereas *z* = 30 retains more discriminative information at the cost of latent continuity. The lower *β* = 0.1 shifts the reconstruction-regularization balance toward decoder fidelity, which is necessary for accurate signal shape reproduction; classification benefits from the stronger regularization of *β* = 1.0.

Cross-species counterfactual decoding (Fig. 6A) was performed by encoding macaque cerebellar neurons (Lisberger; source_id = 3) under their true source label and passing the resulting latent vectors ***μ*** to the decoder under the mouse cerebellar source label (Hausser; source_id = 1). The result is a decoder-predicted signal for what each macaque neuron would look like if recorded in the mouse. The procedure was applied to three shared cell types (GoC, MLI, PkC_ss) across all three modalities.

Latent-space interpolation (Fig. 6D) was performed between a focus macaque GoC neuron and its nearest mouse GoC neighbor in the 16-dimensional latent space (cosine distance). The interpolated vector at step *α* is **z**_*α*_ = (1−*α*) **z**_mac_ + *α* **z**_mouse_ for *α* ∈ {0, 0.25, 0.5, 0.75, 1.0}. Each **z**_*α*_ was decoded under the mouse source label to produce the reconstructed signal at that point along the path.

For the cross-technology interpolation (Supplemental Figs. S5–S8), a separate cVAE was trained jointly on three cortical datasets: A1 (silicon probe; source_id = 0), S1 (juxtacellular micropipette; source_id = 1), and CellExplorer (Neuropixels; source_id = 2). Cell-type labels were standardized before training: PV/FS to Parvalbumin, SOM/SOMnan/SST to Somatostatin, EXC/RS/Pyra to Excitatory. Because A1 and S1 lack ACG data, the ACG modality was set to zero for these datasets, and the model learned to reconstruct it as zero rather than penalizing the encoder. Interpolations followed the same straight-line scheme, with per-class mean latent vectors $\bar{{{\boldsymbol{\mu }}}}$ used in place of individual neuron vectors, decoded under the target dataset’s source label.

#### Data augmentation

Stochastic data augmentations were applied during pretraining and unsupervised fine-tuning; augmentations were disabled during supervised training. Each sample was augmented with probability *p* = 0.3, a light regime selected during the architectural ablation (Fig. 2) over a heavier alternative (*p* = 0.7). Augmentations included additive Gaussian noise (*σ* = 0.03), amplitude scaling (×0.9–1.1), non-linear time warping (warp strength = 0.05), baseline shifts (±0.05), and Gaussian smoothing (*σ* ∈ [0.5, 1.5]), each applied independently with per-type probability 0.3–0.5 and uniformly across all three modalities.

#### Datasets

Juxtacellular Mouse S1 Dataset: Downloaded from the PhysMAP GitHub repository^32^. Pvalb- and Sst-positive interneurons were identified via optotagging in Pvalb-Cre::Ai32 and Sst-Cre::Ai32 mice; post-hoc biocytin fills confirmed cell morphology and cortical layer assignment. The dataset comprises 224 neurons across five classes (L4 Excitatory: 58; L5 Excitatory: 43; L4 Fast-spiking: 35; L5 Fast-spiking: 19; Somatostatin: 69).

Extracellular Mouse Auditory Cortex Dataset (A1): Downloaded from the PhysMAP GitHub repository^31^. Pvalb- and Sst-positive interneurons were identified via optotagging in Pvalb-Cre::Ai32 and Sst-Cre::Ai32 mice using silicon probes; units with ISI violation rate > 2% were excluded. The dataset comprises 285 neurons across three classes (Excitatory: 48; Parvalbumin: 121; Somatostatin: 116).

CellExplorer Dataset (Mouse Visual Cortex and Hippocampus): Downloaded from the PhysMAP GitHub repository. Cell types were identified via optotagging in Pvalb-IRES-Cre::Ai32, Sst-IRES-Cre::Ai32, and Vip-IRES-Cre::Ai32 mice using Neuropixels 1.0 probes^46–48^. The dataset comprises 430 neurons across seven classes (PV: 186; SST: 115; Pyramidal: 44; Axo-axonic: 35; Juxtacellular: 23; VIP: 14; VGAT: 13).

C4 database datasets (Hausser, Hull, and Lisberger): Three datasets were downloaded from the C4 database (https://www.c4-database.com)^23^. Cerebellar cell types were identified via optogenetic tagging (ChR2, GtACR2, or ArchT) under cell-type-specific Cre drivers in C57BL/6J mice and adult male rhesus macaques. Cell types included Purkinje cells (PkC_ss and PkC_cs), MLIs, GoCs, and MFBs.

Hausser (mouse, cerebellar cortex): 1998 total neurons, 104 labeled (GoC: 16; MFB: 13; MLI: 15; PkC_cs: 25; PkC_ss: 35).

Hull (mouse, cerebellar cortex): 103 labeled neurons (GoC: 2; MFB: 18; MLI: 13; PkC_cs: 34; PkC_ss: 36).

Lisberger (macaque, cerebellar floccular complex): 1152 total neurons, 668 expert-labeled (GoC: 188; MFB: 86; MLI: 36; PkC_cs: 147; PkC_ss: 211).

Watson (Rat Neocortex): Extracellular recordings from rat frontal cortex during natural sleep, acquired with 64-site silicon probes (dandiset 000041)^38^. Cell-type labels (Excitatory or Inhibitory) are provided directly in the NWB units table. The dataset comprises 221 neurons (Excitatory: 194; Inhibitory: 27) from 9 subjects. Units were retained with minimum 100 spikes, ISI violations ≤0.5, and presence ratio ≥0.5.

Calvigioni (mouse prefrontal cortex): Extracellular recordings acquired with Neuropixels probes (dandiset 000473)^49^. Neurons were classified as Fast Spiking (Inhibitory) or Regular Spiking (Excitatory) via the NWB units table. The dataset comprises 9213 neurons (Excitatory: 7859; Inhibitory: 1354) from 25 subjects. Units were retained with minimum 100 spikes, ISI violations ≤0.5, and presence ratio ≥0.5.

Ramachandran (rat somatosensory cortex): Extracellular recordings acquired with 32-channel NeuroNexus electrodes (dandiset 000955)^33^. Excitatory and inhibitory neurons were distinguished via CaMKIIα-targeted opsin expression. The dataset comprises 134 neurons (Inhibitory: 105; Excitatory: 29) from a single subject. Only labeled neurons were subset from the NWB.

Allen Institute Visual Coding Dataset: Accessed via AllenSDK. Units were retained using standard quality criteria: ISI violations < 0.5, amplitude cutoff < 0.1, presence ratio > 0.9. The dataset comprises 61,781 neurons from 47 mice spanning 19 Allen Common Coordinate Framework brain regions across visual cortex, hippocampus, and thalamus/midbrain.

IBL Brainwide Map Dataset: Downloaded via the ONE API. Units were retained with IBL quality label good (ISI violations < 0.5, presence ratio ≥0.5, minimum 100 spikes). The dataset comprises 62,993 neurons from 139 subjects and 697 probe insertions across 10 Cosmos-level brain regions.

### Benchmarking methods

1. PCA baselines (PCA-WF, PCA-ISI, PCA-ACG): Linear single-modality baselines that reduce one modality with principal component analysis and classify the result with the shared KNN head. For each modality, we retained the 20 highest-variance input bins, centered and scaled them to unit variance, and projected onto the first five principal components. The reduced dimensionality is not chosen ad hoc: the number of retained features (20) and principal components (5) are exactly the nfeatures and dimV values selected for PhysMAP by the locked hyperparameter sweep on the Hausser dataset (Supplemental Figure S2), so the PCA and PhysMAP baselines operate at matched dimensionality. Principal-component scores were L2-normalized per unit before classification.
2. PhysMAP: PhysMAP integrates multiple physiological modalities via non-linear dimensionality reduction and weighted-nearest neighbor graph construction, inspired by Seurat v4^22^. We used it with waveform, ACG, and ISI modalities; code is available at https://github.com/EricKenjiLee/PhysMAP_Manuscript^21^. Training and test data were passed through PhysMAP to obtain a reduced representation, then fed to the KNN classification head. Hyperparameters (UMAP dimensions dimV ∈ {3, 5, 10, 15}, distance metric ∈ {cosine, euclidean, correlation}, number of features ∈ {20, 40, 80}; 36 configurations) were swept and locked on the Hausser dataset before any other dataset was evaluated (locked configuration: dimV = 5, euclidean metric, nfeatures = 20; KNN balanced accuracy: 0.780).
3. NEMO: NEMO is a contrastive deep learning method that integrates extracellular waveforms and 3D autocorrelograms via a CLIP-like loss^24^; code is available at https://github.com/Haansololfp/NEMO_ICLR. After hyperparameter sweeps on the Hausser dataset (temperature *τ* ∈ {0.1, 0.3, 0.5, 0.7, 1.0}, learning rate ∈ {10^−5^, 10^−4^, 10^−3^}, latent dimensionality *z* ∈ {128, 256, 512}, batch size ∈ {256, 1024}, and weight decay  ∈ {0, 10^−5^, 10^−4^}; 42 configurations selected from this space rather than the full factorial), the locked configuration was: *τ* = 0.5, learning rate 10^−4^, *z* = 256, batch size 1024, up to 200 epochs (locked KNN balanced accuracy: 0.836). No statistical difference was noted with the MLP classification head. Data augmentation was applied stochastically during training: Gaussian noise (probability 0.3, *σ* = 0.1 × std) for waveforms; for ACGs: temporal Gaussian smoothing, temporal jittering, amplitude scaling, additive Gaussian noise, and pepper noise (each with probability 0.5). MLP was used as the main classification head as introduced in the NEMO manuscript. KNN accuracy was reported in the cross-species experiment, the brain region classification task and the hyperparameter tuning section.
4. Unconditioned VAE: An unconditioned variational autoencoder trained on the same modalities and preprocessing as HIPPIE, but without any class or metadata conditioning. The VAE encodes waveform and ISI (bimodal) or waveform, ISI, and ACG (trimodal) into a shared Gaussian latent space and reconstructs all modalities via symmetric decoders. This baseline isolates the contribution of HIPPIE’s conditioning mechanism by holding the generative architecture constant. The latent mean is used as the embedding, fed to the same KNN evaluation head.
5. WF-RF (handcrafted waveform features): A non-deep baseline that extracts nine scalar features from the peak-channel waveform and classifies them with a Random Forest (200 estimators). The four core features follow ref. ^50^: (1) PT ratio (positive peak/absolute negative trough), (2) duration (normalized signed index difference between trough and peak), (3) repolarization slope (amplitude change per sample from trough to peak), and (4) recovery slope (slope from the later extremum to the waveform end). Five additional features follow the Allen Institute ecephys waveform metrics convention: (5) halfwidth (fraction of samples at or above half-maximum absolute amplitude), (6) peak amplitude, (7) trough amplitude, (8) total amplitude (peak–trough), and (9) peak location (index of absolute maximum normalized by waveform length). These nine descriptors are concatenated with PCA components retaining ≥90% of raw waveform variance.
6. HIPPIE 3D + ACG: A variant of HIPPIE in which the standard 1D-ACG branch is replaced with a 3D−ACG branch matching the featurization used by NEMO: the autocorrelogram is computed as a tensor of 10 instantaneous-firing-rate deciles × 100 lag bins (1 ms resolution with 100 ms window) and scaled by a factor of 10 before encoding. All other architectural and conditioning choices are identical to the standard HIPPIE cross-species pipeline. Hyperparameters were latent dimensionality *z* = 100, KL weight *β* = 0.5, batch size 256, 100 epochs, learning rate 3 × 10^−4^ with weight decay 10^−4^ (AdamW with cosine annealing), 5-epoch consistency-regularization warm-up (*λ*_*c*_ = 0.15), and no cross-modal contrastive auxiliary loss.

#### Hyperparameter parity across methods

Matched hyperparameter sweeps were performed for PhysMAP (UMAP dimensions dimV, distance metric, and number of features; 36 configurations) and NEMO (temperature *τ*, learning rate, latent dimensionality *z*, batch size, and weight decay; 42 configurations) on the same Hausser dataset used for HIPPIE hyperparameter selection. The locked configuration for each method was selected by the same stability tiebreaker rule described above and applied to all benchmark datasets without further tuning. The methods were evaluated using equivalent processing pipelines, stratified train/test splits, and the same KNN (*k* selected per method on the training split across *k* ∈ {1, …, 20}) and MLP evaluation heads (for HIPPIE and NEMO; PhysMAP was KNN only). Wall-clock time per cross-validation fold was recorded for HIPPIE, NEMO, PhysMAP, and the baseline VAE on the Hausser dataset; GPU methods ran on NVIDIA GPUs on the NRP Nautilus cluster and CPU-only methods on Intel x86 CPU cores (Supplementary Table S3).

### Negative controls for HIPPIE

We performed negative-control analyses by disrupting the correspondence between samples and class embeddings before classification. Specifically, class embeddings were either randomly shuffled or shifted relative to the input features, while the evaluation labels were kept unchanged. Classification was then repeated on the resulting embeddings.

### Web application and software stack

The web application was built using Streamlit (v1.43.0) and deployed as a browser-based interface for HIPPIE inference. Models were trained offline using PyTorch Lightning and exported as PyTorch checkpoints or ONNX graphs for deployment. Inference uses PyTorch (v2.0.1) and ONNX Runtime (≥1.16.3); core numerical operations rely on NumPy (v1.26.4), SciPy (v1.11.4), and pandas (v1.5.3). NWB file handling uses PyNWB (≥3.1.2), h5py (≥3.10), HDMF (≥4.1.0), and PyArrow (v12.0.1). Additional packages include scikit-learn (v1.2.2) for preprocessing and tqdm (v4.67.1) for progress reporting.

Clustering uses HDBSCAN (v0.8.33) on model-derived embeddings. Dimensionality reduction for visualization uses UMAP (umap-learn v0.5.4), with pynndescent (v0.5.10) and numba (v0.58.1) for efficient nearest-neighbor search and JIT compilation. Interactive visualizations are rendered with Matplotlib (v3.7.1), Bokeh (v2.4.3), Altair (v5.0.1), and PyDeck (v0.9.1). The pretrained checkpoint (hippie_techcond_v1.ckpt) is publicly released on Hugging Face Hub (https://huggingface.co/Jesusgf23/hippie) and downloaded on demand via huggingface_hub (v0.22+) for both web-application inference and programmatic embedding extraction.

### Statistics and reproducibility

This study is a computational re-analysis of previously published, publicly available extracellular electrophysiology datasets; no new animal experiments were performed. The unit of study throughout is the single spike-sorted unit (neuron), and every reported *N* is a count of neurons.

Sample size. No statistical method was used to predetermine sample size. Dataset sizes were fixed by the source publications; we used every unit passing the quality criteria below (*N* = 101–62,993 per benchmark, spanning three orders of magnitude) and report per-fold variability throughout, so estimates from the smaller datasets can be read with appropriate uncertainty.

Data exclusions. Exclusions used pre-established criteria applied before model fitting. (i) Unit quality control followed each source’s standard thresholds: Allen, ISI violations < 0.5, amplitude cutoff < 0.1, presence ratio > 0.9; IBL, the good label (ISI violations < 0.5, presence ratio ≥0.5, ≥100 spikes); DANDI (Watson, Calvigioni, Ramachandran), ≥100 spikes, ISI violations ≤0.5, presence ratio ≥0.5; A1, ISI violation rate ≤2%. (ii) Only ground-truth-labeled units (optotagged or expert-assigned) entered supervised benchmarks; unlabeled units were used only for pretraining. (iii) Classes too small for stratified fivefold splitting were dropped: *n* < 5 for the Hull benchmark (5 classes/103 neurons to 4/101), *n* < 10 for the Hausser dataset (dropping granule cells, *n* = 9; 6 classes/113 neurons to 5/104), and *n* < 10 for the polarity analysis (Supplementary Tables S1 and S2). (iv) Three CellExplorer label groups were not carried into the cell-type analyses: axo-axonic (*n* = 35), juxtacellular (*n* = 23) and VGAT (*n* = 13), reducing that dataset from 7 classes and 430 neurons to 4 classes (parvalbumin, somatostatin, pyramidal, VIP) and 359 neurons. No unit was excluded on the basis of performance or after seeing a result.

Replication. All comparisons were reproduced across the fivefolds of stratified fivefold cross-validation; the fold-to-fold standard deviations report this directly, and no benchmark failed to reproduce. Because the folds resample one fixed set of neurons, they measure estimate stability rather than independent biological replication, and no finding was tested in newly collected recordings. Hyperparameters were fixed on the Hausser dataset before any other benchmark was evaluated, so all remaining benchmarks are out-of-sample with respect to model selection.

Randomization. Experiments were not randomized, and group allocation is not applicable: group membership is the neuron’s ground-truth cell type or brain region, fixed by the source dataset. Fold assignment used stratified pseudo-random partitioning with a fixed seed, identical across methods within a benchmark; animal-level holdout splits (37 training vs. 10 held-out mice for Allen) were fixed before training.

Blinding. Investigators were not blinded, and blinding is not relevant here: all outcome measures (balanced accuracy, macro F1) are computed automatically from held-out ground-truth labels by the shared evaluation script, with no human judgement entering the measurement.

Statistical tests. Balanced accuracy (primary) and macro F1 (secondary) are reported as the mean ± standard deviation across the 5 cross-validation folds. The only inferential analysis, the Pearson correlations between waveform polarity composition and balanced accuracy (Waveform polarity effect analysis, above), reports the correlation coefficient, degrees of freedom, exact *p* value, *R*^2^ effect size, and 95% confidence interval at the point of use. The L4/L5 comparison is descriptive: with only 5 misclassified neurons, it is underpowered, so we report cosine similarities between group-average signals rather than per-bin tests. Method comparisons are reported descriptively with fold-level variability and no significance tests, since folds over one fixed set of neurons are not independent samples and would render such tests anticonservative.

### Reporting summary

Further information on research design is available in the Nature Portfolio Reporting Summary linked to this article.

## Supplementary information

Supplementary Information

Reporting Summary

Transparent Peer Review file

## Source data

Source Data

## Data Availability

All data analyzed in this study are previously published and publicly available; no new data or materials were generated. The Hausser, Hull, and Lisberger cerebellar data used in this study are available in the C4 database (https://www.c4-database.com)^23^. The Juxtacellular Mouse S1^32^, Extracellular Mouse A1^31^, and CellExplorer^46–48^ data used in this study are available in the PhysMAP repository (https://github.com/EricKenjiLee/PhysMAP_Manuscript)^21^. The Watson rat neocortex data used in this study are available in the DANDI Archive under accession code 000041^38^. The Calvigioni mouse prefrontal cortex data used in this study are available in the DANDI Archive under accession code 000473^49^. The Ramachandran rat somatosensory cortex data used in this study are available in the DANDI Archive under accession code 000955^33^. The Allen Institute Visual Coding Neuropixels data used in this study are available in the Allen Brain Observatory via the AllenSDK (https://brain-map.org/our-research/circuits-behavior/visual-coding)^41^. The International Brain Laboratory Brainwide Map data used in this study are available via the ONE API (https://int-brain-lab.github.io/iblenv/notebooks_external/data_release_brainwidemap.html). Source data are provided with this paper.

## Code availability

The user facing code for HIPPIE is available on GitHub: https://github.com/braingeneers/HIPPIE, and the version used in this study has been archived on Zenodo (https://doi.org/10.5281/zenodo.21707907). We have added an extensive tutorial in the shape of a commented Jupyter Notebook. The benchmarking code for the paper release is available in https://github.com/JesusGF1/hippie_benchmarking_release. The pretrained checkpoint (hippie_techcond_v1.ckpt) is publicly available on Hugging Face Hub at https://huggingface.co/Jesusgf23/hippie under the Apache 2.0 license.
