# Supplementary material for: HIPPIE: a generative model for electrophysiological analysis across species, technologies, and modalities
Source: Nat Commun. 2026 Aug 21;17:10007. doi: 10.1038/s41467-026-76939-w (PMC13590638; doi:10.1038/s41467-026-76939-w)
Supplement: Supplementary file 1 [file 41467_2026_76939_MOESM1_ESM.pdf]

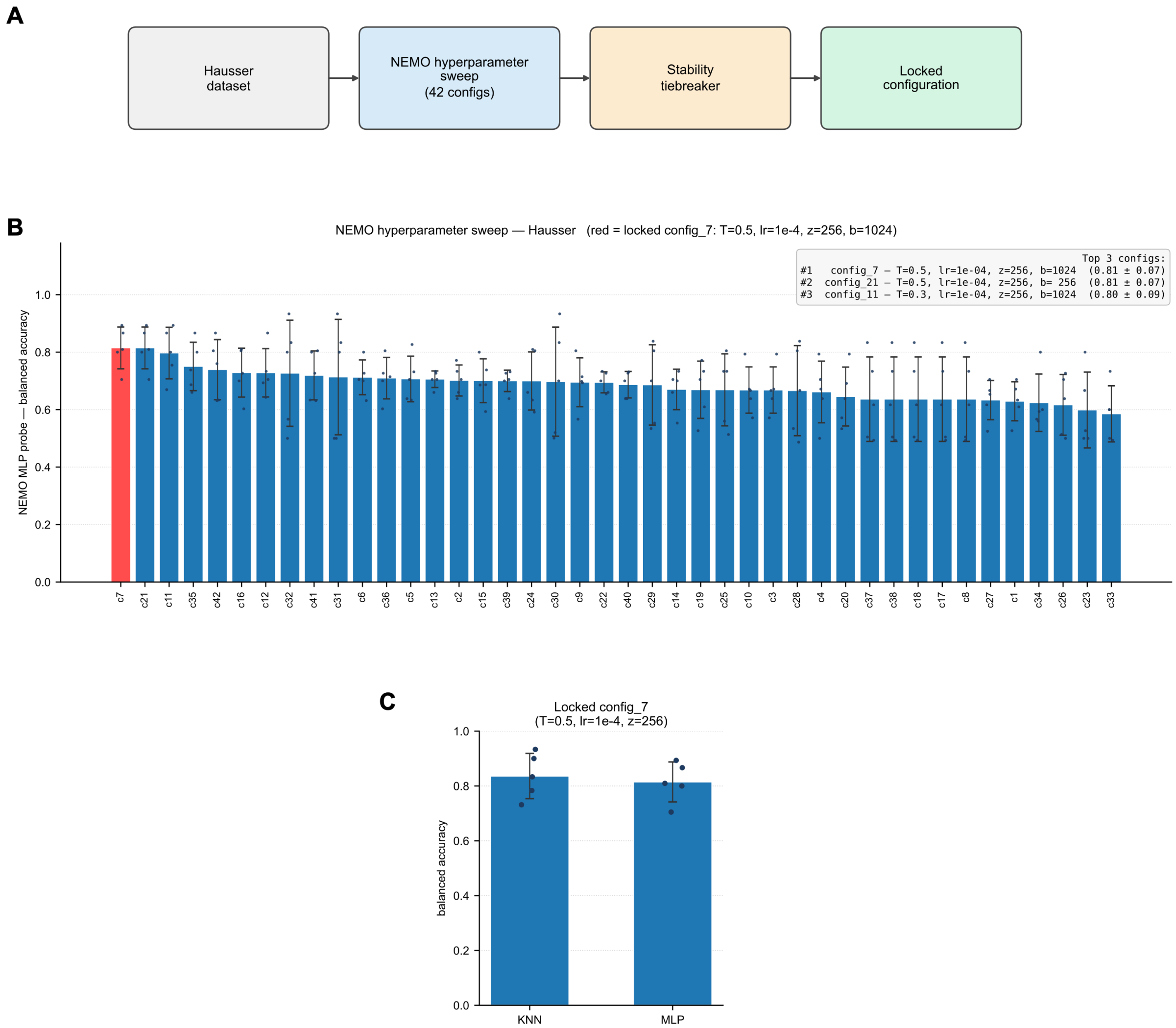

Figure S1. NEMO hyperparameter sweep (Hausser dataset). All panels use the Hausser mouse cerebellar cortex dataset ($N = 104$ labeled neurons, 5 cell types); the unit of study is the single spike-sorted neuron. Balanced accuracy is estimated by stratified 5-fold cross-validation, so every bar is a mean over the $n = 5$ cross-validation folds and every error bar is ± 1 standard deviation across those 5 folds. **A)** NEMO processing pipeline schematic. **B)** Balanced accuracy of the MLP probe, on which the NEMO configuration was selected, across all 42 hyperparameter configurations evaluated on the Hausser dataset, sorted by performance; the locked configuration is highlighted in red. Bars show the mean, error bars ± 1 standard deviation, and overlaid points the 5 individual fold values ($n = 5$ folds). Chance balanced accuracy $= 0.2$. The inset lists the three highest-scoring configurations and their hyperparameters. **C)** KNN vs. MLP probe comparison for the locked NEMO configuration. Bars show the mean, error bars ± 1 standard deviation, and overlaid points the 5 individual fold values ($n = 5$ folds). Source data are provided as a Source Data file.

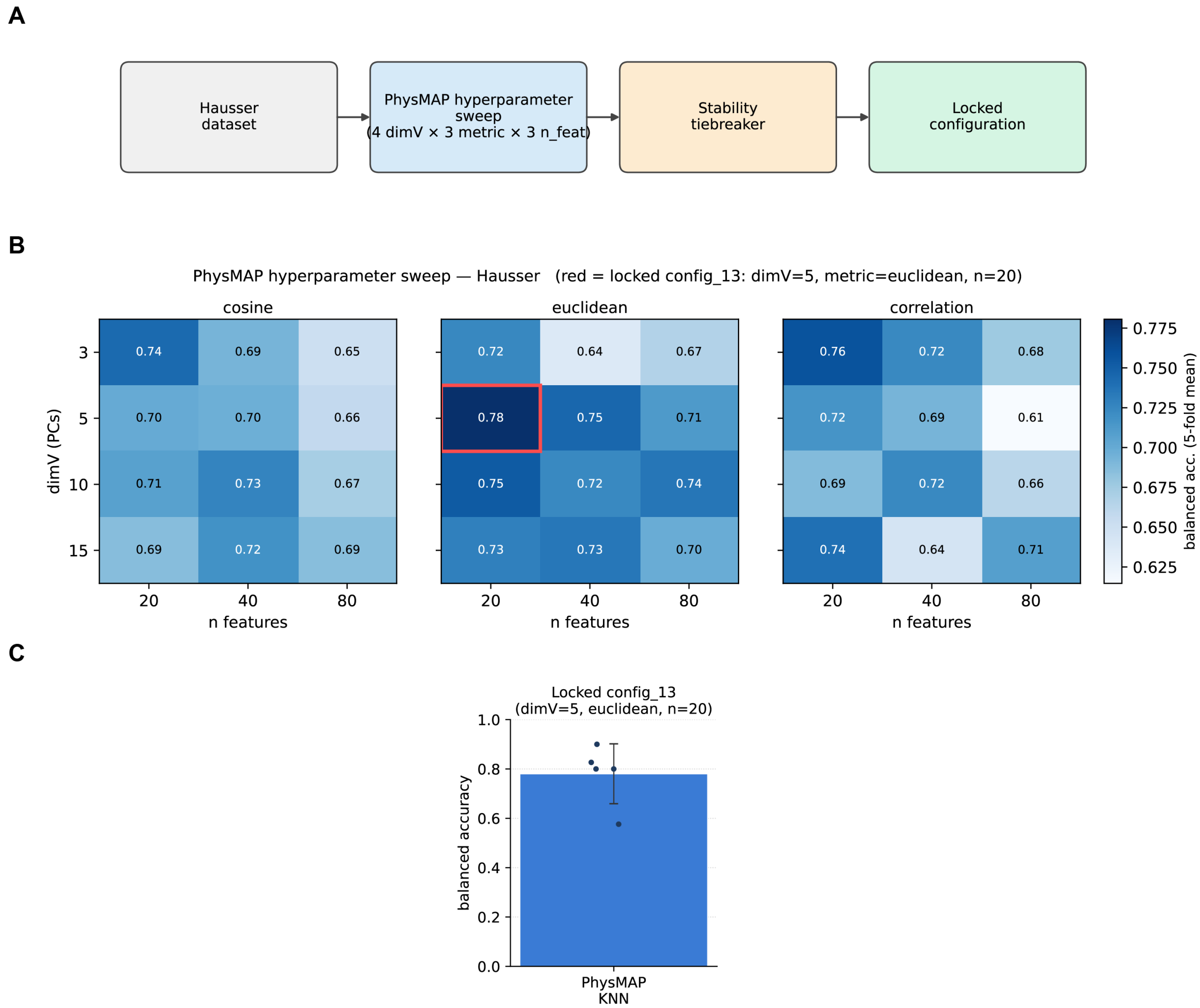

Figure S2. PhysMAP hyperparameter sweep (Hausser dataset). A) PhysMAP processing pipeline schematic. **B)** Balanced accuracy heatmap over reduced-dimension size (`dimV`) and number of features (`nfeatures`) for each evaluation metric. **C)** KNN bar chart for the locked PhysMAP configuration. All panels use the Hausser mouse cerebellar cortex dataset ($N = 104$ labeled neurons, 5 cell types); the unit of study is the single spike-sorted neuron. Heatmap cells in B show the mean balanced accuracy across the $n = 5$ folds of a stratified 5-fold cross-validation; the color scale is given by the accompanying color bar. Bars in C show the mean, error bars ± 1 standard deviation, and overlaid points the 5 individual fold values ($n = 5$ folds). Source data are provided as a Source Data file.

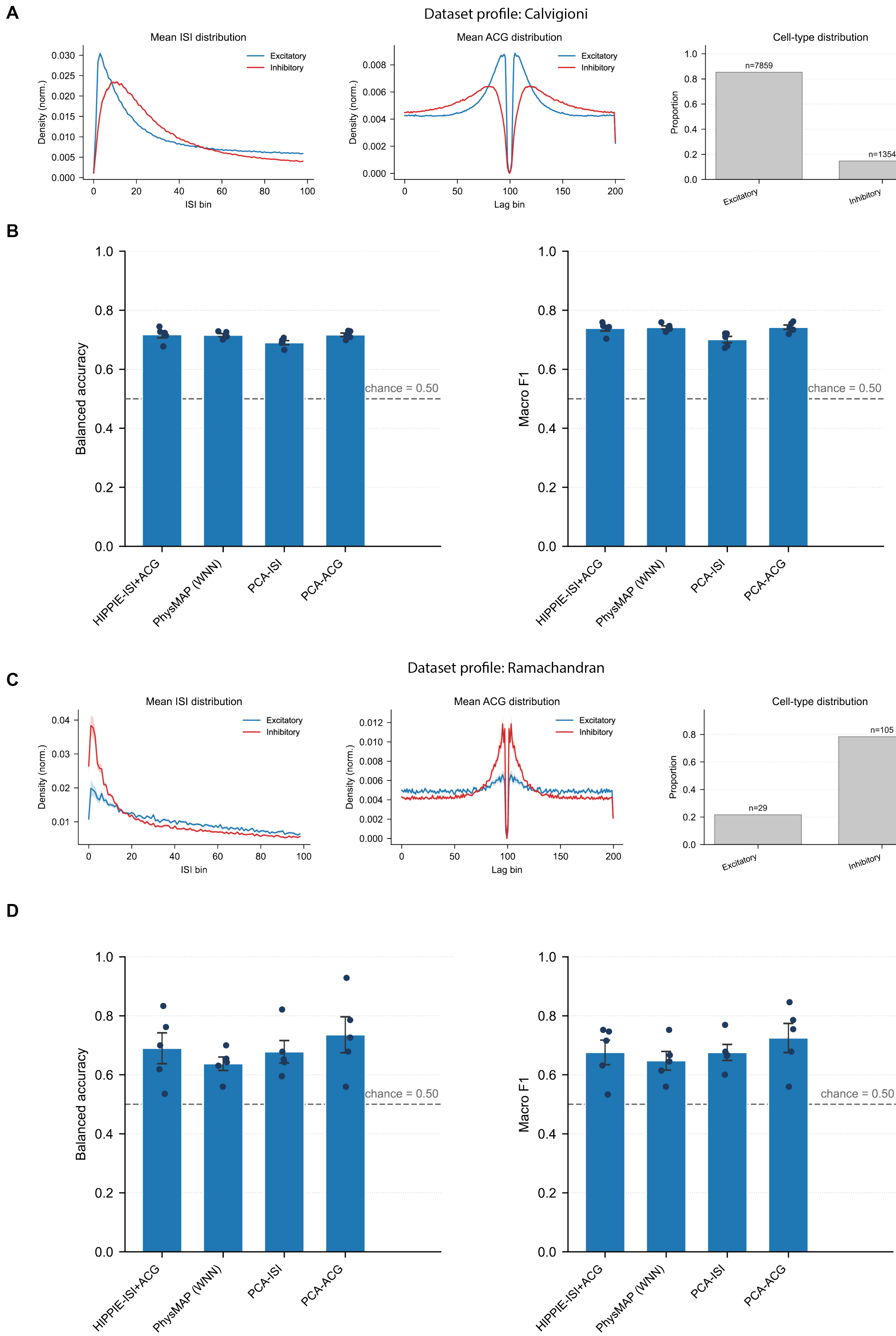

A
Dataset profile: Calvigioni
Mean ISI distribution
Excitatory
Inhibitory
Density (norm.)
ISI bin
Mean ACG distribution
Lag bin
Cell-type distribution
Proportion
n=7859
n=1354
Excitatory
Inhibitory
B
Balanced accuracy
Macro F1
chance = 0.50
HIPPIE-ISI+ACG
PhysMAP (WNN)
PCA-ISI
PCA-ACG
C
Dataset profile: Ramachandran
Mean ISI distribution
Mean ACG distribution
Cell-type distribution
n=29
n=105
D
Balanced accuracy
Macro F1
chance = 0.50

Figure S3. ISI+ACG bimodal benchmarks on Calvigioni and Ramachandran. A) Calvigioni (mouse prefrontal cortex, 9,213 neurons, 2 cell types) electrophysiological profile. **B)** Balanced accuracy comparison Calvigioni; all methods performed comparably (HIPPIE-ISI+ACG: 0.718, PhysMAP: 0.716, PCA-ACG: 0.717, PCA-ISI: 0.690). **C)** Ramachandran (rat somatosensory cortex, 134 neurons, 2 cell types) electrophysiological profile. **D)** Balanced accuracy comparison for Ramachandran; PCA-ACG led (0.736), with larger variance across methods reflecting the small, class-imbalanced dataset (105 inhibitory, 29 excitatory). In all panels the unit of study is the single spike-sorted neuron and N is a count of neurons: Calvigioni $N = 9{,}213$ neurons from 25 subjects (Excitatory 7,859; Inhibitory 1,354); Ramachandran $N = 134$ neurons from 1 subject (Inhibitory 105; Excitatory 29). Chance balanced accuracy $= 0.500$ for both two-class tasks. Bars in B and D show the mean and error bars $\pm$1 standard deviation across the $n = 5$ folds of a stratified 5-fold cross-validation, with overlaid points giving the 5 individual fold values. Feature traces in A and C show the per-cell-type mean $\pm$1 standard deviation across the neurons of that cell type. Abbreviations: ISI = interspike interval distribution; ACG = autocorrelogram; PCA = principal component analysis. Source data are provided as a Source Data file.

A

Watson (Rat Neocortex)

**Species**	Rat
**Region**	Frontal cortex
**Recording**	64-site silicon probe
**N cells**	221
**Cell types**	2
**Reference**	Watson 2016

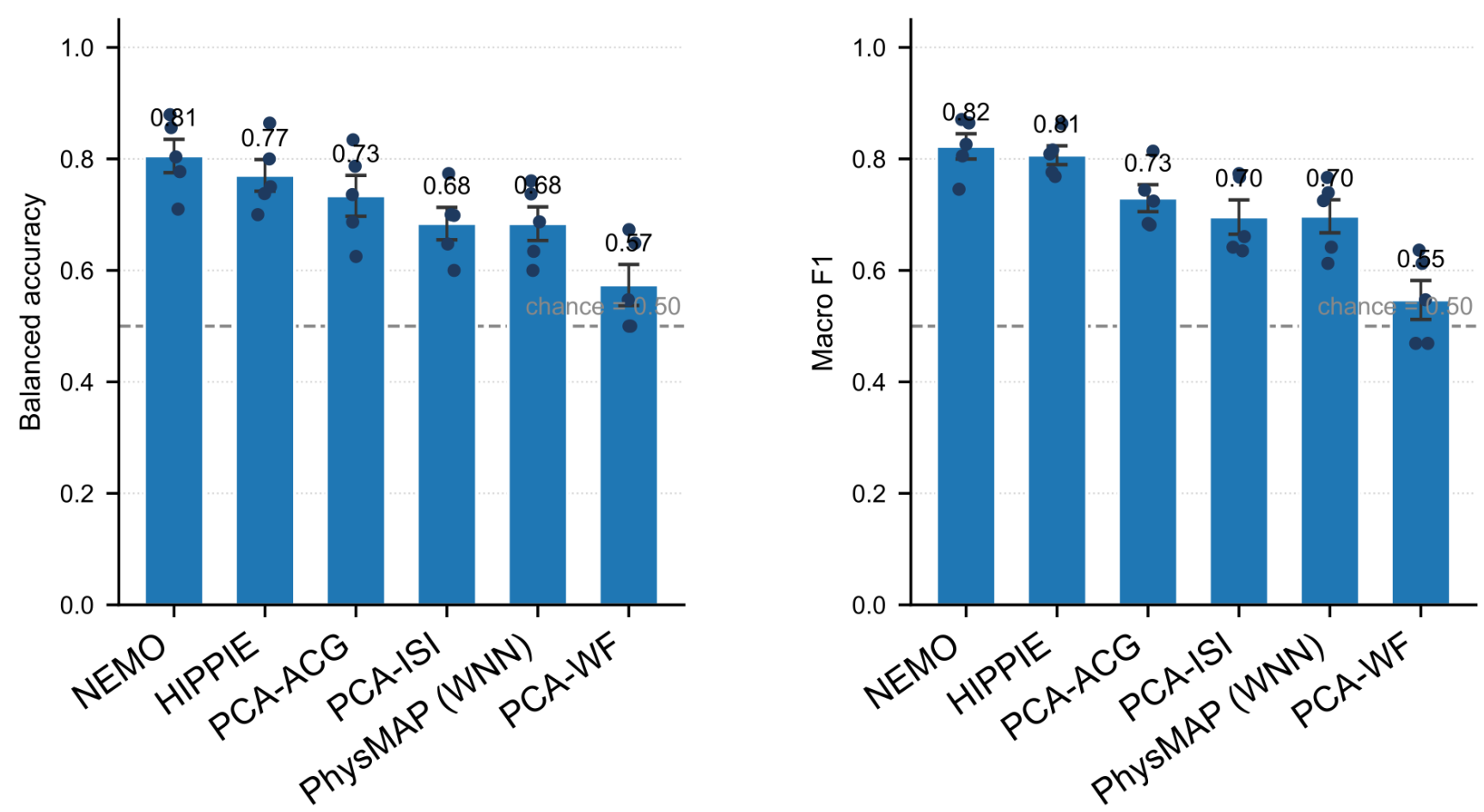

Figure S4. Trimodal benchmark on Watson. Balanced accuracy comparison across methods on the Watson dataset, trimodal cell-type benchmark (221 neurons, 2 cell types, chance = 0.500). All three modalities (waveform, ISI, ACG) were used. The unit of study is the single spike-sorted neuron: $N = 221$ neurons (Excitatory 194; Inhibitory 27). Bars show the mean and error bars ± 1 standard deviation across the $n = 5$ folds of a stratified 5-fold cross-validation, with overlaid points giving the 5 individual fold values. Abbreviations: WF = mean waveform; ISI = interspike interval distribution; ACG = autocorrelogram; PCA = principal component analysis; WF-RF = handcrafted waveform features with a random forest classifier; VAE = unconditioned variational autoencoder. Source data are provided as a Source Data file.

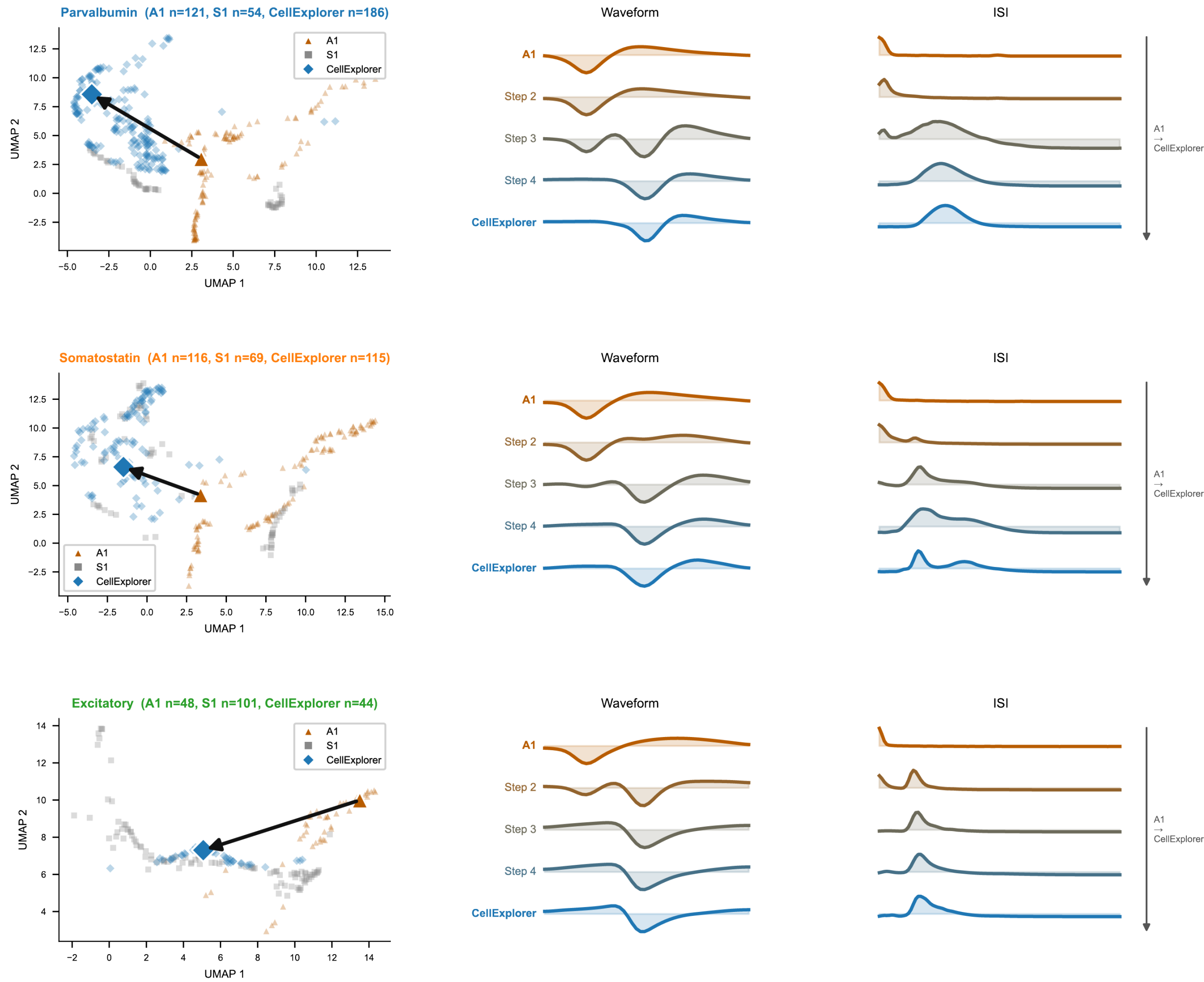

Figure S5. Cross-technology latent-space interpolation (A1 to CellExplorer). 16-D HIPPIE latent space trained jointly on cortical datasets. For each of three cell types (Parvalbumin/fast-spiking, Somatostatin, Excitatory), a focus A1 neuron is interpolated toward its nearest CellExplorer neighbor in 5 steps. Waveform and ISI shown (ACG unavailable for A1 and S1). ISI distributions for CellExplorer neurons appear elongated relative to A1: because CellExplorer uses a shorter ISI computation window, the same absolute inter-spike intervals occupy a larger fraction of the total window, causing the distribution to appear wider and more spread out. The decoder correctly reproduces this dataset-specific ISI shape when decoding under the CellExplorer source label. These panels are decoder outputs for individual neurons rather than derived statistics, and are reproducible from the deposited checkpoint and code, so no source data file accompanies this figure.

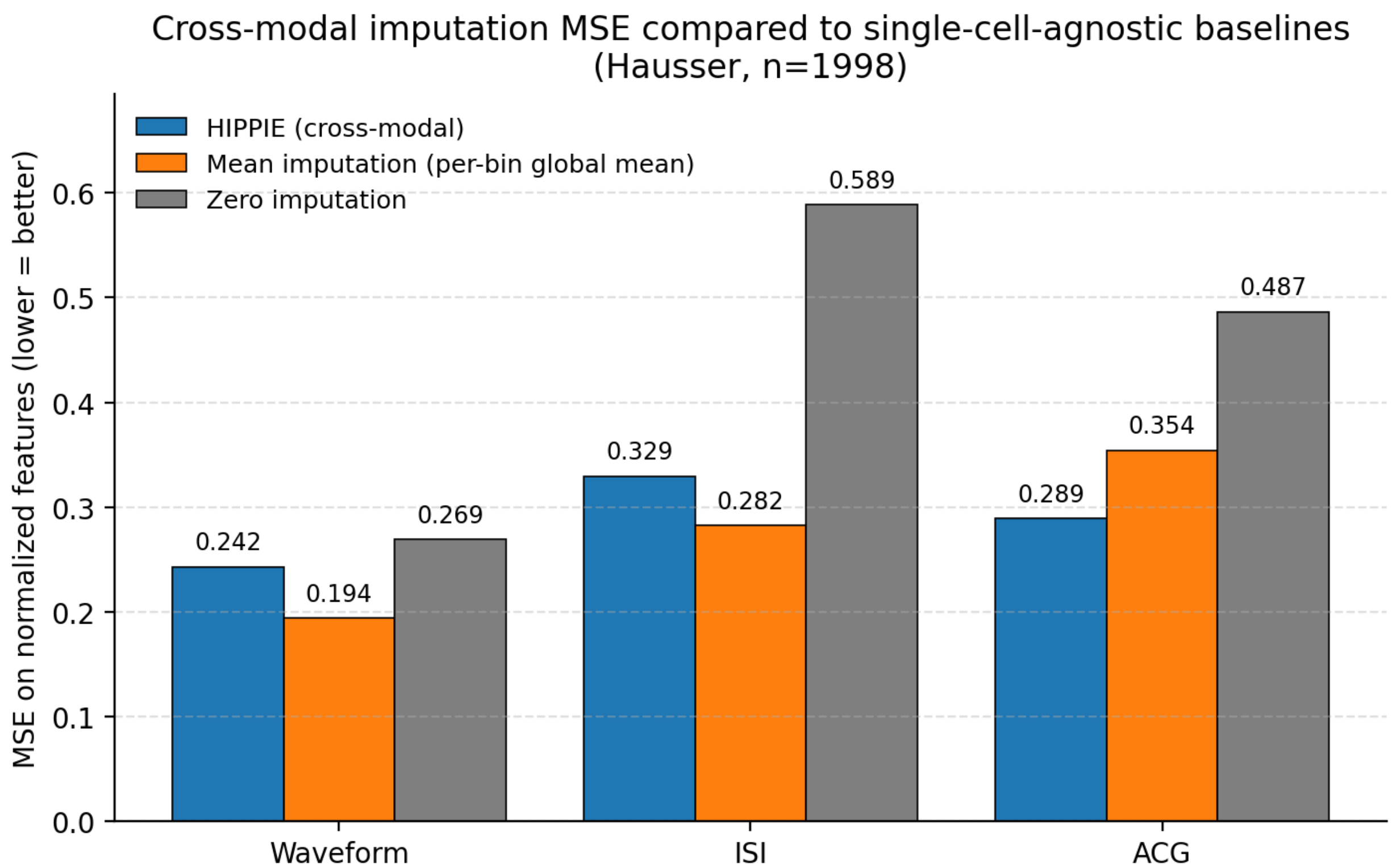

Figure S6. Cross-modal imputation anchored against single-cell-agnostic baselines (Hausser). For each modality, HIPPIE's self-imputation MSE (blue, diagonal entries of Figure 6B) is compared against two non-model baselines that use no cell-specific information: mean imputation (orange), which predicts the per-bin population mean of the masked modality, and zero imputation (grey), which predicts zero. Beating the mean baseline indicates that the unmasked modalities carry cell-specific information about the masked one. HIPPIE beats both baselines for ACG (0.289 vs. 0.354 vs. 0.487), is close to the mean baseline for ISI (0.329 vs. 0.282 vs. 0.589), and slightly underperforms it for waveform (0.242 vs. 0.194 vs. 0.269), identifying ACG as the modality with the strongest cross-modal recovery and waveform morphology as the most informationally independent modality, as discussed in the main text. Source data are provided as a Source Data file.

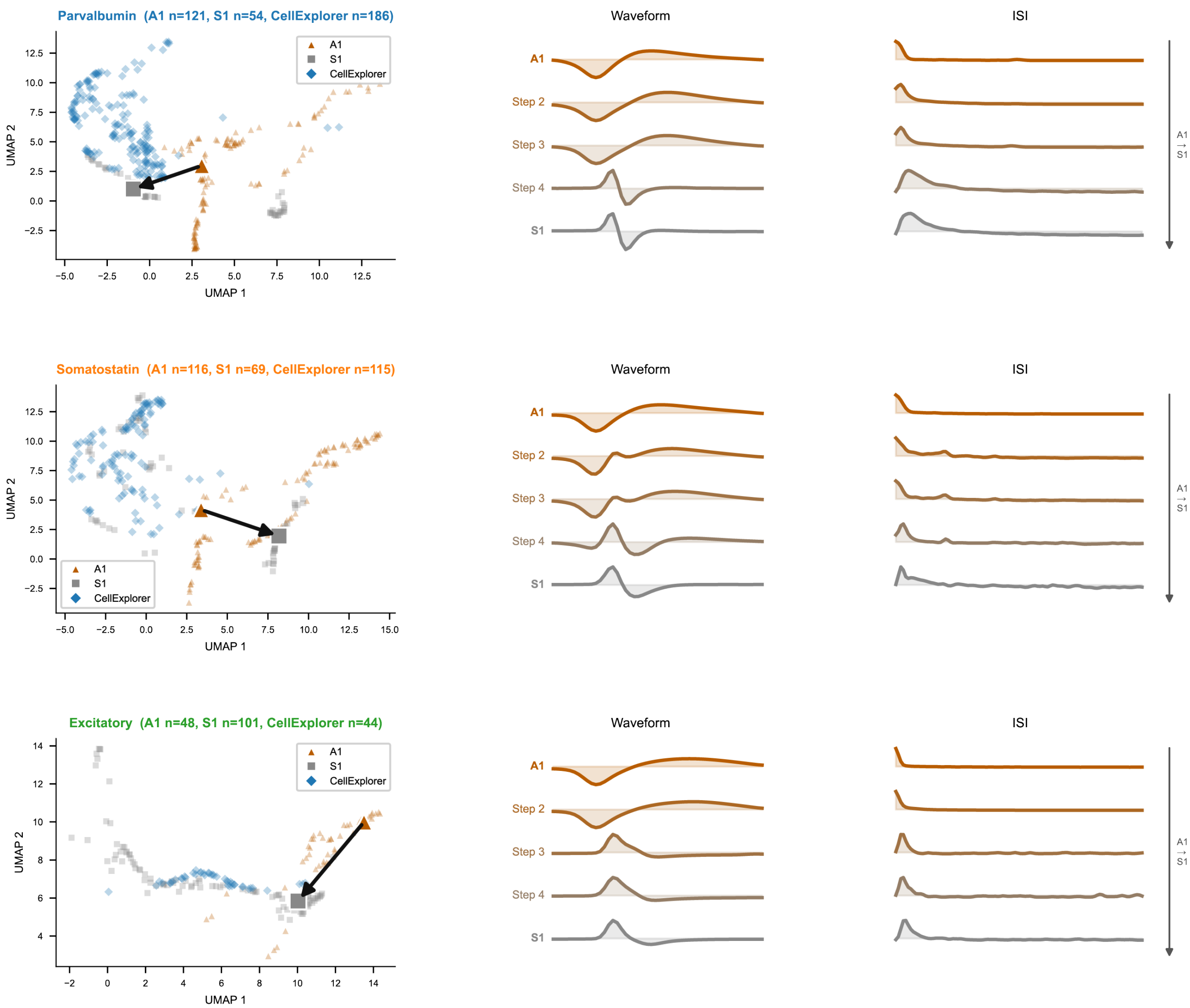

Figure S7. Cross-technology latent-space interpolation (A1 to S1). Same layout as Supplemental Figure S5. Waveform morphology transitions smoothly from A1 to S1 across all three cell types. These panels are decoder outputs for individual neurons rather than derived statistics, and are reproducible from the deposited checkpoint and code, so no source data file accompanies this figure.

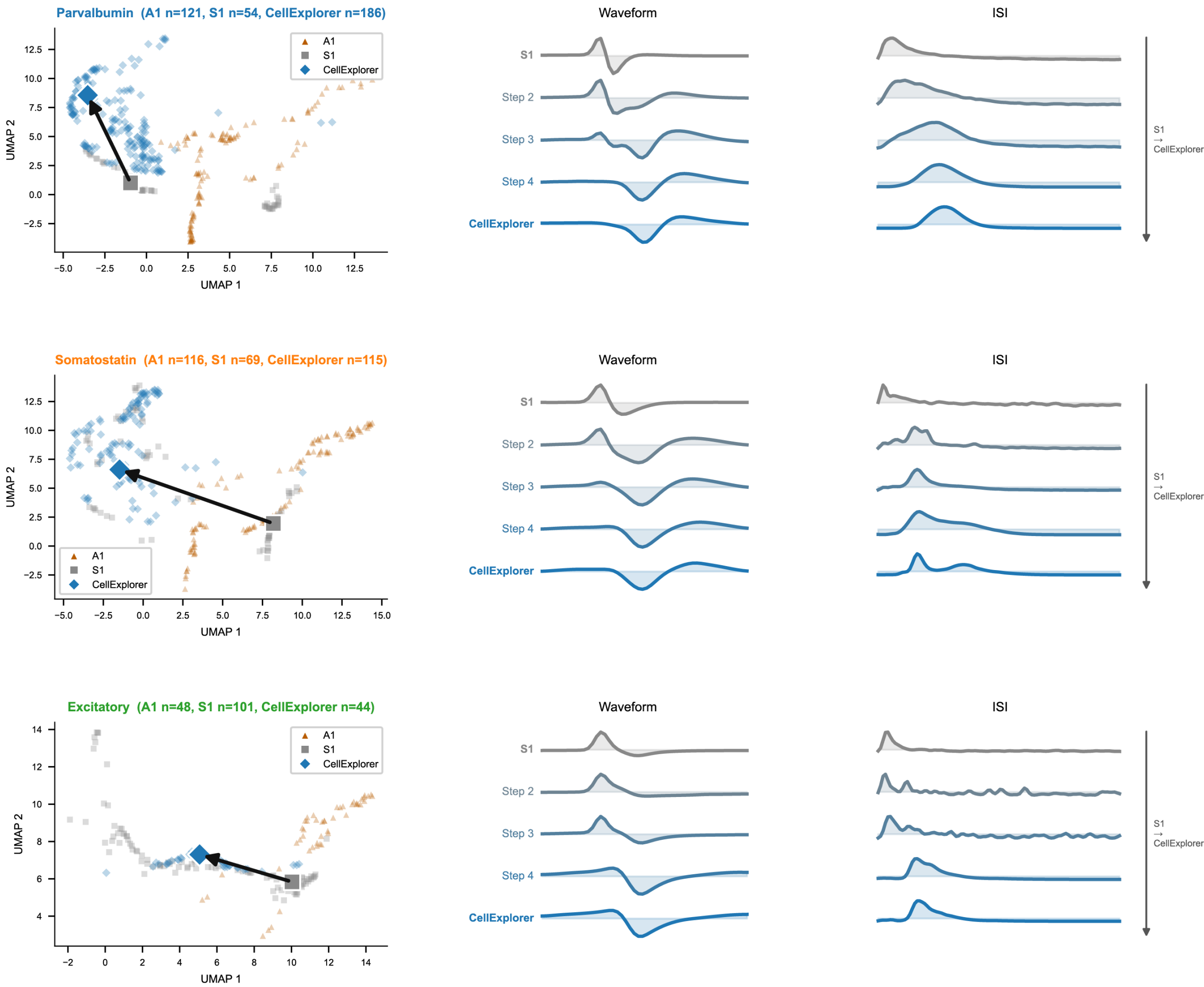

Figure S8. Cross-technology latent-space interpolation (S1 to CellExplorer). Same layout as Supplemental Figure S5. The elongated ISI shape characteristic of CellExplorer is again reproduced by the decoder at the CellExplorer endpoint. These panels are decoder outputs for individual neurons rather than derived statistics, and are reproducible from the deposited checkpoint and code, so no source data file accompanies this figure.

Table S1. Waveform polarity composition and HIPPIE classification accuracy across nine cell-type datasets. N **is the number of labeled neurons after rare-class filtering (**$n < 10$ **per class). Bal. Acc is the mean balanced accuracy (KNN, transductive)** $\pm$ **standard deviation across 5 folds. Because rare classes are removed here, these values can differ from the corresponding main-text accuracies.**

Dataset	N	% negative	% biphasic	% positive	Bal. Acc	SD
Hull	101	55.4	38.6	5.9	0.949	0.039
Lisberger	668	55.1	7.9	37.0	0.678	0.027
Watson	221	89.1	10.0	0.9	0.770	0.057
Calvigioni	9213	98.0	1.9	0.1	0.718	0.022
Ramachandran	134	0.0	100.0	0.0	0.690	0.105
Hausser	104	54.8	42.3	2.9	0.682	0.066
Juxtacellular S1	224	0.0	45.5	54.5	0.914	0.051
A1 auditory	285	42.1	57.9	0.0	0.569	0.065
CellExplorer	430	63.5	36.5	0.0	0.534	0.035
Pearson r vs. Bal. Acc		-0.18	-0.10	$+0.45$		

Table S2. Within-dataset balanced accuracy stratified by waveform polarity group (HIPPIE transductive KNN, pooled across 5 folds). Overall is the aggregate balanced accuracy across all test neurons; polarity-stratified rows show accuracy restricted to test neurons in each polarity class, sorted by descending accuracy.

Dataset	Polarity	N (test)	Bal. Acc
Hull	Overall	101	0.945
	negative	56	0.953
	biphasic	39	0.940
	positive	6	0.750
Lisberger	Overall	668	0.678
	positive	247	0.650
	negative	368	0.613
	biphasic	53	0.572
Hausser	Overall	104	0.677
	biphasic	44	0.602
	negative	57	0.599
CellExplorer	Overall	359	0.534
	negative	253	0.541
	biphasic	106	0.507

Table S3. Wall-clock time per cross-validation fold (Hausser cell-type dataset). Methods are sorted by runtime. PCA-based methods are near-instantaneous (< 1 s). Data loading time is not tracked separately and is included in the pretrain/training phase.

Method	Modalities	Time/fold (min)	Hardware	Folds
PCA-WF	WF	< 0.02	CPU	1
PCA-ISI	ISI	< 0.02	CPU	1
PCA-ACG	ACG	< 0.02	CPU	1
PhysMAP	WF+ISI+ACG	0.36	CPU (R)	1
Baseline VAE	WF+ISI+ACG	1.2 ± 0.5	GPU	5
NEMO	WF+ACG	1.5 ± 0.4	GPU	5
HIPPIE	WF+ISI+ACG	1.8 ± 0.3	GPU	5

HIPPIE phase breakdown (mean): pretrain 1.2 min, finetune 0.3 min, supervised 0.1 min, embedding + KNN 0.2 min.
