# Supplementary material for: HIPPIE: a generative model for electrophysiological analysis across species, technologies, and modalities
Source: Nat Commun. 2026 Aug 21;17:10007. doi: 10.1038/s41467-026-76939-w (PMC13590638; doi:10.1038/s41467-026-76939-w)
Supplement: Supplementary file 2 [file 41467_2026_76939_MOESM2_ESM.pdf]

nature portfolio

Corresponding author(s): Mohammed Mostajo-Radji

Last updated by author(s): Aug 1, 2026

Reporting Summary

Nature Portfolio wishes to improve the reproducibility of the work that we publish. This form provides structure for consistency and transparency in reporting. For further information on Nature Portfolio policies, see our Editorial Policies and the Editorial Policy Checklist.

Statistics

For all statistical analyses, confirm that the following items are present in the figure legend, table legend, main text, or Methods section.

n/a	Confirmed	
☐	☒	The exact sample size (*n*) for each experimental group/condition, given as a discrete number and unit of measurement
☐	☒	A statement on whether measurements were taken from distinct samples or whether the same sample was measured repeatedly
☐	☒	The statistical test(s) used AND whether they are one- or two-sided<br>*Only common tests should be described solely by name; describe more complex techniques in the Methods section.*
☐	☒	A description of all covariates tested
☒	☐	A description of any assumptions or corrections, such as tests of normality and adjustment for multiple comparisons
☐	☒	A full description of the statistical parameters including central tendency (e.g. means) or other basic estimates (e.g. regression coefficient) AND variation (e.g. standard deviation) or associated estimates of uncertainty (e.g. confidence intervals)
☐	☒	For null hypothesis testing, the test statistic (e.g. *F*, *t*, *r*) with confidence intervals, effect sizes, degrees of freedom and *P* value noted<br>*Give P values as exact values whenever suitable.*
☒	☐	For Bayesian analysis, information on the choice of priors and Markov chain Monte Carlo settings
☒	☐	For hierarchical and complex designs, identification of the appropriate level for tests and full reporting of outcomes
☒	☐	Estimates of effect sizes (e.g. Cohen's *d*, Pearson's *r*), indicating how they were calculated

Our web collection on statistics for biologists contains articles on many of the points above.

Software and code

Policy information about availability of computer code

Data collection	N/A
Data analysis	HIPPIE, the software described in this paper. THe code has been deposited in https://github.com/braingeneers/HIPPIE and https://doi.org/10.5281/zenodo.21707907

For manuscripts utilizing custom algorithms or software that are central to the research but not yet described in published literature, software must be made available to editors and reviewers. We strongly encourage code deposition in a community repository (e.g. GitHub). See the Nature Portfolio guidelines for submitting code & software for further information.

Data

Policy information about availability of data

All manuscripts must include a data availability statement. This statement should provide the following information, where applicable:

- Accession codes, unique identifiers, or web links for publicly available datasets
- A description of any restrictions on data availability
- For clinical datasets or third party data, please ensure that the statement adheres to our policy

All data used for this study was downloaded from existing datasets.

Research involving human participants, their data, or biological material

Policy information about studies with human participants or human data. See also policy information about sex, gender (identity/presentation), and sexual orientation and race, ethnicity and racism.

Reporting on sex and gender	N/A
Reporting on race, ethnicity, or other socially relevant groupings	N/A
Population characteristics	N/A
Recruitment	N/A
Ethics oversight	N/A

Note that full information on the approval of the study protocol must also be provided in the manuscript.

Field-specific reporting

Please select the one below that is the best fit for your research. If you are not sure, read the appropriate sections before making your selection.

[x] Life sciences [ ] Behavioural & social sciences [ ] Ecological, evolutionary & environmental sciences

For a reference copy of the document with all sections, see nature.com/documents/nr-reporting-summary-flat.pdf

Life sciences study design

All studies must disclose on these points even when the disclosure is negative.

Sample size	No statistical method was used to predetermine sample size. Dataset sizes were fixed by the source publications; we used every unit passing the quality criteria below (N = 101-62,993 per benchmark, spanning three orders of magnitude) and report per-fold variability throughout so estimates from the smaller datasets can be read with appropriate uncertainty.
Data exclusions	xclusions used pre-established criteria applied before model fitting. (i) Unit quality control followed each source's standard thresholds: Allen, ISI violations <0.5, amplitude cutoff <0.1, presence ratio >0.9; IBL, the good label (ISI violations <0.5, presence ratio ≥ 0.5, ≥ 100 spikes); DANDI (Watson, Calvigioni, Ramachandran), ≥ spikes, ISI violations ≤ 0.5, presence ratio ≥ 0.5; A1, ISI violation rate >2%. (ii) Only ground-truth-labeled units (optotagged or expert-assigned) entered supervised benchmarks; unlabeled units were used only for pretraining. (iii) Classes too small for stratified 5-fold splitting were dropped: n < 5 for the Hull benchmark (5 classes/103 neurons to 4/101)
Replication	We created a complementary codebase to ensue the reproducibility of all the data-based figures in the paper. All data was replicated correctly using our pipelines
Randomization	Experiments were not randomized, and group allocation is not applicable: group membership is the neuron's ground-truth cell type or brain region, fixed by the source dataset. Fold assignment used stratified pseudo-random partitioning with a fixed seed, identical across methods within a benchmark; animal-level holdout splits (37 training vs.10 held-out mice for Allen) were fixed before training
Blinding	Investigators were not blinded, and blinding is not relevant here: all outcome measures (balanced accuracy, macro F1) are computed automatically from held-out ground-truth labels by the shared evaluation script, with no human judgement entering the measurement.

Reporting for specific materials, systems and methods

We require information from authors about some types of materials, experimental systems and methods used in many studies. Here, indicate whether each material, system or method listed is relevant to your study. If you are not sure if a list item applies to your research, read the appropriate section before selecting a response.

Materials & experimental systems

n/a	Involved in the study	
[x]	[ ]	Antibodies
[x]	[ ]	Eukaryotic cell lines
[x]	[ ]	Palaeontology and archaeology
[x]	[ ]	Animals and other organisms
[x]	[ ]	Clinical data
[x]	[ ]	Dual use research of concern
[x]	[ ]	Plants

Methods

n/a	Involved in the study	
[x]	[ ]	ChIP-seq
[x]	[ ]	Flow cytometry
[x]	[ ]	MRI-based neuroimaging

Plants

Seed stocks	N/A
Novel plant genotypes	N/A
Authentication	N/A
