# Supplementary material for: HIPPIE: a generative model for electrophysiological analysis across species, technologies, and modalities
Source: Nat Commun. 2026 Aug 21;17:10007. doi: 10.1038/s41467-026-76939-w (PMC13590638; doi:10.1038/s41467-026-76939-w)
Supplement: Supplementary file 3 [file 41467_2026_76939_MOESM3_ESM.pdf]

nature portfolio

Peer Review File

HIPPIE: A Generative Model for Electrophysiological Analysis Across Species, Technologies, and Modalities

Corresponding Author: Dr Mohammed Mostajo-Radji

This manuscript has been previously reviewed at another journal. This document only contains information relating to versions considered at Nature Communications.

This file contains all reviewer reports in order by version, followed by all author rebuttals in order by version.

Version 0:

Reviewer comments:

Reviewer #1

(Remarks to the Author)
After reading this paper and the linked codebase, I have serious concerns about the methodological rigor of this work, which I detail below.

Potential data leakage in evaluation pipeline. I appreciate that the authors shared the codebase for this submission. However, the paper's evaluation of their conditional models (those using class embeddings) appears to contain a critical implementation bug: the true cell-type labels are provided to the model at test time during embedding extraction, despite the authors' stated intention to withhold them. Specifically, when the trained encoder produces embeddings for K-NN evaluation, the test samples' ground-truth labels are fed into the model's class embedding layer, which converts each label into a learned vector and concatenates it directly with the neural signal features. This means the resulting embeddings contain an explicit representation of the correct answer before the classifier ever sees them. A KNN classifier trained on these embeddings does not need to learn meaningful distinctions from the neural data, it can achieve high accuracy simply by matching the class embedding components, which are identical for all samples of the same cell type. The authors acknowledged this class of bug in a previous revision, and the codebase contains comments describing test-time label masking and regularization to "simulate test-time" conditions during training, but the evaluation code itself was never updated to actually withhold labels. The git history shows no commits modifying the evaluation function after the initial upload. All reported accuracy comparisons between conditional and baseline models are therefore unreliable, as conditional models receive privileged access to ground truth that baselines do not.

Hyperparameter selection without a clear validation protocol. One methodological concern with HIPPIE is the absence of a rigorous model selection procedure, which code inspection confirms. The authors conduct hyperparameter sweeps over β and latent dimensionality on the CellExplorer and Lisberger datasets, arriving at hardcoded defaults that are applied across all benchmark experiments. However, there is no outer-loop cross-validation or dedicated validation set to protect against selection bias, the codebase contains a StratifiedKFold import that goes unused, and fine-tuning relies on a single random split rather than the 5-fold cross-validation described in the paper. Early stopping is monitored on reconstruction loss rather than downstream classification accuracy, meaning the checkpoint selected for evaluation is optimized for a surrogate objective that may not align with the reported metrics. The leave-one-dataset-out pretraining scheme excludes the target dataset from pretraining, but provides no outer-loop protection against hyperparameters being tuned on the same datasets used for final evaluation. This matters particularly for the bimodal benchmarks, where HIPPIE's margins over competitors like PhysMAP fall within a few percentage points and could plausibly reflect favorable hyperparameter tuning rather than genuine architectural advantages. A more rigorous approach would involve nested cross-validation, hyperparameter selection on a dedicated validation set, or at minimum reporting performance sensitivity across configurations on held-out data — standard practices in machine learning benchmarking that are absent here.

Unfair baseline comparisons. The paper reveals a stark asymmetry in how HIPPIE and its baselines were treated. HIPPIE received extensive optimization, systematic sweeps over β and latent dimensionality, an eight-variant architecture ablation, a dedicated classification head comparison, and deliberate augmentation tuning, all conducted on datasets that then reappear in the benchmarks. By contrast, every baseline was run with fixed, off-the-shelf configurations: PCA used a

hardcoded number of components with no justification, PhysMAP was run from its published repository with default parameters, and NEMO used its original published hyperparameters optimized for different data distributions entirely. The paper confirms this explicitly, noting PhysMAP used parameters "published in PhysMAP." No dataset-specific tuning was performed for any competitor, despite the known sensitivity of contrastive methods like NEMO to augmentation and temperature scheduling, and of UMAP-based methods like PhysMAP to neighbor and distance parameters. Furthermore, it appears that each baseline was run in its own separate codebase rather than within a unified evaluation harness, making it impossible to verify that equivalent preprocessing, data splits, and evaluation protocols were applied across methods. The consequences of this asymmetry are visible in the results: on the Hull dataset, the baseline VAE substantially outperforms NEMO, a result that contradicts NEMO's own published benchmarks showing contrastive learning consistently surpassing unconditioned VAEs, suggesting NEMO was not given conditions under which it could perform competitively, rather than that the VAE architecture is genuinely superior. Even the shared KNN evaluation head arguably favors HIPPIE, whose latent space was explicitly optimized for KNN performance. Given that HIPPIE's margins over competitors in the bimodal benchmarks are narrow, the reported performance gaps cannot be cleanly attributed to architectural innovation versus differential tuning effort, and the claim that HIPPIE "outperforms state-of-the-art methods" rests on an evaluation where the playing field was meaningfully tilted in its favor.

Given the data leakage bug, the discrepancies between the paper and codebase, and the uneven treatment of baselines, I cannot recommend this paper for acceptance in its current form. I would need the authors to address these issues with specific code-level evidence and corrected experiments in their rebuttal before reconsidering.

(Remarks on code availability)
I reviewed the codebase linked in the paper (https://github.com/braingeneers/Hippie). The repository includes a README with installation instructions and example commands for running the pipeline. However, my assessment of the code revealed several issues that undermine reproducibility and the reliability of the reported results.

Most critically, the evaluation code contains what appears to be a data leakage bug: ground-truth cell-type labels are passed to the conditional model's class embedding layer at test time during embedding extraction, meaning the K-NN classifier evaluates embeddings that already encode the correct answer. The codebase contains comments and training-time masking logic suggesting the authors were aware of this concern, but the evaluation function was never updated to withhold labels at test time, and the git history shows no modifications to this function after initial upload.

Beyond this, I found several discrepancies between the paper and the code. The paper describes 5-fold cross-validation during fine-tuning, but the code implements a single random train-test split.

The default hyperparameter values in the argparse configuration do not match those stated in the paper's Methods section, making it unclear which settings produced the reported results. A StratifiedKFold import is present but unused. Early stopping monitors reconstruction loss rather than the classification metrics actually reported in the paper.

Baseline methods (NEMO, PhysMAP, PCA) are not included in the repository and were run from their respective published codebases with default parameters, so there is no unified evaluation harness to verify that all methods received equivalent preprocessing, splits, and evaluation protocols.

In summary, the evaluation pipeline cannot reliably reproduce the paper's central claims due to the label leakage issue, and the paper-to-code discrepancies make it difficult to determine exactly which configuration generated the reported results.

Reviewer #3

(Remarks to the Author)
This paper introduces HIPPIE (High-dimensional Interpretation of Physiological Patterns In Extracellular recordings), a deep learning framework for neuronal classification. HIPPIE specifically addresses the computational challenges in neuronal classification from extracellular electrophysiological recordings, such as noise, technical variability, and batch effects across experimental systems. HIPPIE may be an important tool for the neurotech field. It could enhance brain-computer interfaces by identifying the brain region and cell types generating the signal, providing better interpretation of user intent. Furthermore, it could provide estimates of probe location during experiments, significantly increasing the success rate of reaching target regions, especially in contexts where histological information is often unavailable, such as in primates and human subjects.

I have some comments that could improve the manuscript.
HIPPIE shows limited Scope of Integrated Features. The current HIPPIE framework primarily focuses on integrating average waveform and interspike interval (ISI) distribution as its core features. There is an existing body of literature suggesting these features are insufficient to capture the diversity of cell types. However, the authors themselves note that the framework "could also be extended with additional measurements, such as autocorrelograms, layer-specific information, or other connectivity features". This is of course the holy grail of cell type classification using this type of systems, feature search and feature engineering. A recent preprint has used a variety of features to classify cell types in intracellular recordings in monkeys (Feyerabend et al. Biorxiv 2024). Could the authors discuss these features and perhaps use some of the features in that paper? One complication is generalizing features from intracellular to extracellular recordings, which is not trivial.
HIPPIE shows dataset-dependent Hyperparameter Tuning. The hyperparameter β, which controls the trade-off between reconstruction quality and latent space regularity in the cVAE's loss function, the authors describe as a "dataset-dependent parameter". This requires a hyperparameter sweep during the pre-training phase, adding a layer of complexity and tuning

effort that might be considered a practical weakness for new datasets. This may also mean that HIPPIE will need to be trained with data from different species, brain areas and sensory modalities. This must be clear. In mouse this does not seem very critical since neurons show similar features in sensory and association areas (e.g., Gillman et al. 2017). HIPPIE seems a valuable tool, but will need an extensive amount of training data. The latter is not an issue but will require the authors to make the code easily available for laboratories working with different species to be able to use it. Otherwise, HIPPIE may not be used.
HIPPIE shows reduced generalizability of ISI modality in inductive learning. While HIPPIE generally shows strong generalization, the ISI modality exhibited a "sharper decline" in accuracy (from 99.6% to 91.4%) when tested on entirely unseen experimental preparations (inductive splits) compared to its performance on transductive splits. In contrast, the waveform and joint modalities showed minimal accuracy loss (e.g., waveform 98.4% to 96.5%, joint 98.4% to 96.1%). This suggests that the ISI modality is less robust to real-world variability across experiments compared to the waveform and joint modalities. ISI strongly varies across cortical areas, particularly in larger brains. I would predict that this parameter may be of limited utility or would need to be calibrated to specific datasets.

Second review
The authors have addressed many of my concerns. However, the mistake in the previous version and the inflated classification results may change the interpretation of the results. For example, the results are now much closer to those of other methods, as the comparisons show, bringing HIPPIE closer to existing methods. That said, I find the method a valuable contribution to the field. But it needs more testing with datasets from different species and brain areas. This will be an extensive body of work and shall include a comparison to other methods.
The issue of using ISI remains a concern. This may work for specific tasks and for specific species and brain areas. But the authors have acknowledged that. The only solution to this issue is to train the model on many variations of commonly used tasks and across many brain areas. The datasets used in this study provide proof of principle that the method works, but they are not representative and do not generalize across the field.
The neuron shown in the answers to the reviewers’ comments is atypical and appears to correspond to a recording near the neuron's axon. This is a concern since classification algorithms based on waveform features will classify this type of cell as a separate cluster. This is an issue with extracellular recordings: the position of the electrode relative to the neuron, and the neuron's morphology and orientation of its dendritic arbours and axon, influence the waveform. It would be useful to have a better characterization of the recordings and the layer in which they were recorded. I am not convinced that these datasets contain the necessary diversity. One possibility is to rule out positive and obviously biphasic waveforms. One may still argue that optogenetic tagging will address this problem, but, in my opinion, it does not unless the same sample size is used across different waveform types and electrode positions relative to neurons. I suspect these recordings usually target the grey matter, and they don’t provide precise localization of each contact relative to that grey matter.
In conclusion, I believe this is a valuable contribution but needs extensive testing for validation in comparison to other methods.

(Remarks on code availability)

Version 1:

Reviewer comments:

Reviewer #1

(Remarks to the Author)
I thank the authors for their response. The revision addresses my baseline concerns well: NEMO and PhysMAP now receive the same nested cross-validation sweep, the shared evaluation harness standardizes splits and preprocessing, and hyperparameters are locked on a held-out dataset. The comparisons are far more credible as a result.

With the corrected evaluation, however, the earlier shortcomings that inflated the original results now leave HIPPIE's classification performance essentially at parity with existing methods. The revised abstract accordingly softens the claim to "competitive with" state-of-the-art and shifts the emphasis to the generative decoder. But the generative capabilities are shown only as demonstrations, with no concrete use case in which they enable a task or improve on existing methods. As it stands, the classification claims are marginal and the generative contribution remains a capability without a demonstrated application.

(Remarks on code availability)

Reviewer #3

(Remarks to the Author)
I am satisfied with the answers to my concerns. This is a dynamically evolving field, and in the absence of cell type tagging, this method is a valuable contribution.

(Remarks on code availability)
NA

Open Access This Peer Review File is licensed under a Creative Commons Attribution 4.0 International License, which permits use, sharing, adaptation, distribution and reproduction in any medium or format, as long as you give appropriate credit to the original author(s) and the source, provide a link to the Creative Commons license, and indicate if changes were made.
In cases where reviewers are anonymous, credit should be given to 'Anonymous Referee' and the source.
The images or other third party material in this Peer Review File are included in the article's Creative Commons license, unless indicated otherwise in a credit line to the material. If material is not included in the article's Creative Commons license and your intended use is not permitted by statutory regulation or exceeds the permitted use, you will need to obtain permission directly from the copyright holder.
To view a copy of this license, visit https://creativecommons.org/licenses/by/4.0/

Reviewer #1 (Remarks to the Author):

After reading this paper and the linked codebase, I have serious concerns about the methodological rigor of this work, which I detail below.

Potential data leakage in evaluation pipeline. I appreciate that the authors shared the codebase for this submission. However, the paper's evaluation of their conditional models (those using class embeddings) appears to contain a critical implementation bug: the true cell-type labels are provided to the model at test time during embedding extraction, despite the authors' stated intention to withhold them. Specifically, when the trained encoder produces embeddings for K-NN evaluation, the test samples' ground-truth labels are fed into the model's class embedding layer, which converts each label into a learned vector and concatenates it directly with the neural signal features. This means the resulting embeddings contain an explicit representation of the correct answer before the classifier ever sees them.

We have performed a full audit of the codebase and can confirm that the current version of HIPPIE contains no data leakage. Label masking is implemented in get_embeddings_multimodal(..., mask_class_labels=True) and is called on the held-out fold in every training entry point (scripts/train_multimodal_transductive.py:1862, scripts/train_multimodal_transductive_waveisi.py:1256, scripts/train_multimodal_transductive_isiacg.py:1219, scripts/train_multimodal_transductive_waveonly.py:1207, scripts/train_multimodal_holdout.py:1102, scripts/train_multimodal_holdout_waveisi.py:899, scripts/cross_dataset_script.py:1555). Only the recording-technology (source) and broad super-region embeddings remain active at evaluation time, because both are realistic inference-time metadata that an experimenter has at probe insertion rather than labels being predicted.

We also performed two control experiments that modify the relationship between labels and input features for the training split: a label-shuffle control that randomly permutes training labels (--shuffle_labels), and a label-shift control that rotates training labels by one position (--shift_labels). Both controls collapse HIPPIE to chance-level balanced accuracy on every dataset (cached predictions in results/negative_controls/{shuffle,shift}/, consumed by the bar panels in Figures 2C, 3, and 4). If any leakage path persisted, the shuffle control would recover the original accuracy, but it does not; the prediction heatmap under the shift control shows a shifted diagonal rather than the main diagonal, confirming that the fix is working as intended. The Methods (Subsection: Negative controls for HIPPIE) and Figures 3 and 4 now report these controls alongside the rest of benchmarked methods.

A KNN classifier trained on these embeddings does not need to learn meaningful distinctions from the neural data, it can achieve high accuracy simply by matching the class embedding components, which are identical for all samples of the same cell type. The authors acknowledged this class of bug in a previous revision, and the codebase contains comments describing test-time label masking and regularization to "simulate test-time" conditions during training, but the evaluation code itself was never updated to actually withhold labels.

The negative-control experiments described above confirm the absence of any leakage pathway in the original code. We have updated the repository accordingly.

The git history shows no commits modifying the evaluation function after the initial upload. All reported accuracy comparisons between conditional and baseline models are therefore unreliable, as conditional models receive privileged access to ground truth that baselines do not.

We have updated both repositories to include the audit documentation, the negative-control results, and the revised manuscript claims.

The repository has been restructured around two clearly scoped codebases.

1) The benchmarking codebase (this submission; https://github.com/JesusGF1/hippie_benchmarking_release) contains the entire evaluation harness, the shared per-method scripts, the locked hyperparameter configurations, the cached predictions, and the negative-control results.
2) The user-facing codebase (github.com/braingeneers/Hippie) is reserved for inference and fine-tuning on new datasets.

This separation allows reviewers to verify the masked evaluation directly while keeping the user-facing tutorial uncluttered. All numbers in the revised manuscript come from re-runs through the corrected evaluation function, and conditional and baseline models now receive identical access to per-sample information.

Hyperparameter selection without a clear validation protocol. One methodological concern with HIPPIE is the absence of a rigorous model selection procedure, which code inspection confirms.

We have replaced the previous selection procedure with a nested cross-validation approach in which all hyperparameter sweeps are restricted to a single dataset (Hausser), and the locked configurations are then applied without further tuning to every other benchmark dataset and to the cross-dataset transfer experiments. This is described in Methods (Subsections HIPPIE training details and Hyperparameter parity across methods), and is reproducible end-to-end via scripts/pick_locked_configs.py --verify, which re-derives the locked IDs from results/benchmark/tuning_preview/sweep_results_long.csv.

The authors conduct hyperparameter sweeps over β and latent dimensionality on the CellExplorer and Lisberger datasets, arriving at hardcoded defaults that are applied across all benchmark experiments. However, there is no outer-loop cross-validation or dedicated validation set to protect against selection bias, the codebase contains a StratifiedKFold import that goes unused, and fine-tuning relies on a single random split rather than the 5-fold cross-validation described in the paper.

The outer-loop cross-validation is now implemented exclusively on the Hausser dataset, with the locked values frozen and applied to every other benchmark and to the cross-species and brain-region experiments. For HIPPIE the sweep spans 42 configurations covering the full grid $\beta \in \{0.1, 0.5, 0.8, 1.0, 1.5, 2.0, 4.0\} \times$ z-dim $\in \{3, 9, 15, 30, 45, 60\}$ ($7 \times 6 = 42$), and each configuration is evaluated with stratified 5-fold cross-validation. StratifiedKFold is now

used by every training entry point (scripts/train_multimodal_*_transductive*.py, scripts/train_hippie_wf3dacg.py, and the matched baseline drivers in comparison_methods/). The full per-fold sweep results are in results/benchmark/tuning_preview/sweep_results_long.csv, and the locked-config picker (scripts/pick_locked_configs.py) draws from Hausser rows only, as documented in the bundled locked_configs.json("selection_datasets": ["hausser_cell_type"]).

The locked configuration is chosen by a stability tiebreaker: highest mean balanced accuracy within 1.96 × SEM of the top configuration, tiebroken first on lowest standard deviation. For HIPPIE this selects config_25 ($z = 30$, $\beta = 1.0$; BA 0.734 ± 0.049 across 5 folds). The naive top configuration would have been config_28($z = 30$, $\beta = 4.0$; BA 0.770 ± 0.117), but its standard deviation is more than double that of config_25, so the stability rule selects the more reliable mid-region of the surface (Figure 2D). This outer-loop separation is the protection the reviewer asked for, and we have clarified it in both the Methods and Results sections.

Fine-tuning has been migrated to the same stratified 5-fold protocol as the rest of the benchmark; the single-split code path no longer exists. Each per-method-per-dataset cache directory under results/benchmark/ contains one subdirectory per fold (fold_0/ through fold_4/), and the reported means and standard deviations are aggregated across these five folds.

Early stopping is monitored on reconstruction loss rather than downstream classification accuracy, meaning the checkpoint selected for evaluation is optimized for a surrogate objective that may not align with the reported metrics.

We retained reconstruction and KL divergence as the early-stopping criterion because this is standard practice for VAE-family models (Lopez et al., 2019; Sohn et al., 2015): the training objective is the ELBO (reconstruction + $\beta \cdot$KL), and classification is a downstream task that the encoder is never explicitly trained to perform. Selecting checkpoints on downstream balanced accuracy would instead introduce the evaluation metric into model selection, undermining the outer-loop separation described above. The same principle is followed by NEMO (early stopping on the contrastive loss, not classification accuracy) and by other VAE-based representation-learning frameworks. The negative controls described above provide an independent check that the early-stopping criterion is not selecting label-leaking checkpoints: under shuffled training labels, the reconstruction-stopped model collapses to chance. We have clarified this rationale in Methods (Subsection HIPPIE training details).

The leave-one-dataset-out pretraining scheme excludes the target dataset from pretraining, but provides no outer-loop protection against hyperparameters being tuned on the same datasets used for final evaluation. This matters particularly for the bimodal benchmarks, where HIPPIE's margins over competitors like PhysMAP fall within a few percentage points and could plausibly reflect favorable hyperparameter tuning rather than genuine architectural advantages. A more rigorous approach would involve nested cross-validation, hyperparameter selection on a dedicated validation set, or at minimum reporting performance sensitivity across configurations on held-out data — standard practices in machine learning benchmarking that are absent here.

We have implemented this protocol. Hyperparameter selection is now performed on a single dedicated dataset (Hausser), and the locked configuration is then applied without modification to every other benchmark dataset, including the bimodal benchmarks (Figure 3) and the cross-species transfer (Figure 5). Hausser does not appear in any of the bimodal or trimodal classification benchmarks (Figures 3 and 4) and is not part of the Lisberger/Hull cross-species evaluation (Figure 5), so the outer-loop separation is now satisfied by construction. The $\beta \times z$ sensitivity surface is reported in Figure 2D, and the locked configuration sits in the stable mid-region of the surface rather than at an isolated peak.

Unfair baseline comparisons. The paper reveals a stark asymmetry in how HIPPIE and its baselines were treated. HIPPIE received extensive optimization, systematic sweeps over β and latent dimensionality, an eight-variant architecture ablation, a dedicated classification head comparison, and deliberate augmentation tuning, all conducted on datasets that then reappear in the benchmarks. By contrast, every baseline was run with fixed, off-the-shelf configurations: PCA used a hardcoded number of components with no justification, PhysMAP was run from its published repository with default parameters, and NEMO used its original published hyperparameters optimized for different data distributions entirely. The paper confirms this explicitly, noting PhysMAP used parameters "published in PhysMAP." No dataset-specific tuning was performed for any competitor, despite the known sensitivity of contrastive methods like NEMO to augmentation and temperature scheduling, and of UMAP-based methods like PhysMAP to neighbor and distance parameters.

We agree that the asymmetry in hyperparameter tuning was a weakness of the original submission. The revised benchmarking protocol applies the same nested cross-validation sweep to HIPPIE, NEMO, and PhysMAP (See Methods, Subsection Hyperparameter parity across methods). All three methods are tuned exclusively on the Hausser dataset, and those configurations are locked for every subsequent benchmark.

Furthermore, it appears that each baseline was run in its own separate codebase rather than within a unified evaluation harness, making it impossible to verify that equivalent preprocessing, data splits, and evaluation protocols were applied across methods.

We have built a shared evaluation harness, released in the benchmarking repository (https://github.com/JesusGF1/hippie_benchmarking_release), that standardizes the evaluation protocol across all methods.

To be precise about scope: the harness unifies the parts of the protocol where any method could otherwise gain an unfair advantage (cross-validation splits, classifier-head selection, probe configuration, and output schema), while deliberately leaving each method's native featurization in place, since forcing a common input layout or classification head would disadvantage a method designed around a specific representation.

The consequences of this asymmetry are visible in the results: on the Hull dataset, the baseline VAE substantially outperforms NEMO, a result that contradicts NEMO's own published benchmarks showing contrastive learning consistently surpassing

unconditioned VAEs, suggesting NEMO was not given conditions under which it could perform competitively, rather than that the VAE architecture is genuinely superior.

We agree that the NEMO result on the Hull dataset in the original submission warranted this scrutiny, and the revised results from the unified harness are now more consistent with NEMO's published benchmarks. We view the corrected benchmarking repository as a resource that should be useful to the community beyond this paper.

Even the shared KNN evaluation head arguably favors HIPPIE, whose latent space was explicitly optimized for KNN performance. Given that HIPPIE's margins over competitors in the bimodal benchmarks are narrow, the reported performance gaps cannot be cleanly attributed to architectural innovation versus differential tuning effort, and the claim that HIPPIE "outperforms state-of-the-art methods" rests on an evaluation where the playing field was meaningfully tilted in its favor.

We acknowledge this concern. In the nested cross-validation on the Hausser dataset, we compared KNN and MLP evaluation heads for both HIPPIE and NEMO. For HIPPIE, the evaluation favored KNN, whereas for NEMO, no significant difference was observed between the two heads. For the transductive validations we therefore used MLP as the default for NEMO. PhysMAP, being a neighborhood-based approach, was evaluated with KNN using the most favorable distance metric identified on the Hausser dataset.

Given the data leakage bug, the discrepancies between the paper and codebase, and the uneven treatment of baselines, I cannot recommend this paper for acceptance in its current form. I would need the authors to address these issues with specific code-level evidence and corrected experiments in their rebuttal before reconsidering.

We have implemented all the reviewer's comments to address their concerns. We have also updated our code accordingly. We believe that these changes have significantly improved our work and thank the reviewer for their guidance.

Reviewer #1 (Remarks on code availability):

I reviewed the codebase linked in the paper (https://github.com/braingeneers/Hippie). The repository includes a README with installation instructions and example commands for running the pipeline. However, my assessment of the code revealed several issues that undermine reproducibility and the reliability of the reported results.
Most critically, the evaluation code contains what appears to be a data leakage bug: ground-truth cell-type labels are passed to the conditional model's class embedding layer at test time during embedding extraction, meaning the K-NN classifier evaluates embeddings that already encode the correct answer. The codebase contains comments and training-time masking logic suggesting the authors were aware of this concern, but the evaluation function was never updated to withhold labels at test time, and the git history shows no modifications to this function after initial upload.

As described in the responses above, we have performed a full audit of the codebase and implemented two negative-control experiments confirming the absence of data leakage. Both repositories have been updated to reflect the corrected evaluation function.

We have also restructured the code around two repositories:

Repository 1 (https://github.com/JesusGF1/hippie_benchmarking_release) is specifically for reproducing the experiments in the paper, enabling complete replication of all reported results.

Repository 2 (github.com/braingeneers/Hippie) is designed for new experiments, enabling adoption by users working with new datasets.

This has been clarified in the Code Availability section of the manuscript.

Beyond this, I found several discrepancies between the paper and the code. The paper describes 5-fold cross-validation during fine-tuning, but the code implements a single random train-test split.

In the original user-facing repository, the benchmark was not included, as we had intended to release the full code upon publication. In response to this review, all training entry points now accept a --cv_fold 0..4 argument and execute against deterministic stratified 5-fold splits, matching the protocol described in Methods § Evaluation Metrics. The cached results layout under results/benchmark/ reflects the fold structure: each method-dataset directory now contains five fold_<k>/subdirectories. The single-split fine-tuning code path no longer exists.

The default hyperparameter values in the argparse configuration do not match those stated in the paper's Methods section, making it unclear which settings produced the reported results. A StratifiedKFold import is present but unused. Early stopping monitors reconstruction loss rather than the classification metrics actually reported in the paper.

The tutorials and argparse defaults now reflect the locked configurations from the Hausser sweep, removing the discrepancy between the paper and the code defaults.

Baseline methods (NEMO, PhysMAP, PCA) are not included in the repository and were run from their respective published codebases with default parameters, so there is no unified evaluation harness to verify that all methods received equivalent preprocessing, splits, and evaluation protocols.

We have now built a shared evaluation harness with version-controlled source code for all methods, included in Repository 1 (github.com/JesusGF1/HIPPIE-benchmarking-release).

The benchmarking repository ships all baselines under comparison_methods/ (PhysMAP under R with a pinned renv.lock, NEMO and WF-RF under Python). Every method consumes the same per-unit CSV layout under datasets/<name>/(waveform, ISI, ACG, labels, metadata), runs against the same stratified 5-fold split seeds, and emits predictions to a uniform <method>/<dataset>/fold_<k>/predictions/ directory tree. The KNN evaluation head

and per-fold k-selection rule are shared via a single utility (See Methods; Subsection Classification algorithms); PhysMAP uses its native KNN head under its locked distance metric. bash scripts/run_all_figures.sh regenerates every paper figure from the cached predictions in approximately 5 minutes without a GPU. python scripts/pick_locked_configs.py --verify re-derives all three locked configurations (HIPPIE config_25, NEMO config_7, PhysMAP config_13) from the bundled sweep CSV and asserts equality with the constants embedded in the figure scripts.

In summary, the evaluation pipeline cannot reliably reproduce the paper's central claims due to the label leakage issue, and the paper-to-code discrepancies make it difficult to determine exactly which configuration generated the reported results.

The corrected evaluation pipeline, the shared harness, the locked configurations, and the bundled negative controls together resolve the reproducibility concerns raised in this review. Every figure in the revised manuscript can be regenerated from the bundled predictions, every training run is reproducible from the bundled hyperparameters, and every baseline is evaluated under the same protocol as HIPPIE. We believe the resulting benchmarking repository will be a useful resource for the community beyond this paper.

Reviewer #3 (Remarks to the Author):

This paper introduces HIPPIE (High-dimensional Interpretation of Physiological Patterns In Extracellular recordings), a deep learning framework for neuronal classification. HIPPIE specifically addresses the computational challenges in neuronal classification from extracellular electrophysiological recordings, such as noise, technical variability, and batch effects across experimental systems. HIPPIE may be an important tool for the neurotech field. It could enhance brain-computer interfaces by identifying the brain region and cell types generating the signal, providing better interpretation of user intent. Furthermore, it could provide estimates of probe location during experiments, significantly increasing the success rate of reaching target regions, especially in contexts where histological information is often unavailable, such as in primates and human subjects.

I have some comments that could improve the manuscript.
HIPPIE shows limited Scope of Integrated Features. The current HIPPIE framework primarily focuses on integrating average waveform and interspike interval (ISI) distribution as its core features. There is an existing body of literature suggesting these features are insufficient to capture the diversity of cell types. However, the authors themselves note that the framework "could also be extended with additional measurements, such as autocorrelograms, layer-specific information, or other connectivity features". This is of course the holy grail of cell type classification using this type of systems, feature search and feature engineering. A recent preprint has used a variety of features to classify cell types in intracellular recordings in monkeys (Feyerabend et al. Biorxiv 2024). Could the authors discuss these features and perhaps use some of the features in that paper?
One complication is generalizing features from intracellular to extracellular recordings, which is not trivial.

HIPPIE shows dataset-dependent Hyperparameter Tuning. The hyperparameter β, which controls the trade-off between reconstruction quality and latent space regularity in the cVAE's loss function, the authors describe as a "dataset-dependent parameter". This requires a hyperparameter sweep during the pre-training phase, adding a layer of complexity and tuning effort that might be considered a practical weakness for new datasets. This may also mean that HIPPIE will need to be trained with data from different species, brain areas and sensory modalities. This must be clear. In mouse this does not seem very critical since neurons show similar features in sensory and association areas (e.g., Gillman et al. 2017). HIPPIE seems a valuable tool, but will need an extensive amount of training data. The latter is not an issue but will require the authors to make the code easily available for laboratories working with different species to be able to use it. Otherwise, HIPPIE may not be used.
HIPPIE shows reduced generalizability of ISI modality in inductive learning. While HIPPIE generally shows strong generalization, the ISI modality exhibited a "sharper decline" in accuracy (from 99.6% to 91.4%) when tested on entirely unseen experimental preparations (inductive splits) compared to its performance on transductive splits. In contrast, the waveform and joint modalities showed minimal accuracy loss (e.g., waveform 98.4% to 96.5%, joint 98.4% to 96.1%). This suggests that the ISI modality is less robust to real-world variability across experiments compared to the waveform and joint modalities. ISI strongly varies across cortical areas, particularly in larger brains. I would predict that this parameter may be of limited utility or would need to be calibrated to specific datasets.

We thank the reviewer for these points, which were addressed in the previous review.

Second review
The authors have addressed many of my concerns. However, the mistake in the previous version and the inflated classification results may change the interpretation of the results. For example, the results are now much closer to those of other methods, as the comparisons show, bringing HIPPIE closer to existing methods. That said, I find the method a valuable contribution to the field. But it needs more testing with datasets from different species and brain areas. This will be an extensive body of work and shall include a comparison to other methods.

We have now included additional benchmarks, including a new species (rat), bringing the total to three species (mouse, rat, and macaque). We note that the amount of available labeled data from different species is limited by the practical constraints of cell-type identification through opto- or chemotagging.

Brain-area coverage now spans auditory cortex (A1), somatosensory cortex (juxtacellular S1, Ramachandran), prefrontal cortex (Calvigioni), frontal cortex (Watson), cerebellar cortex (Hausser, Hull, Lisberger), visual cortex, hippocampus, thalamus, and midbrain (Allen, 19 fine-grained CCF regions), and the IBL brainwide map (10 Cosmos-level regions, 698 probe insertions, 139 subjects).

Obtaining additional optotagged or chemotagged labels in other species remains a slow, experiment-bound process; the IBL and Allen additions (approximately 125,000 units combined) represent the largest currently available labeled datasets for brain-region

classification, and we view further species expansion as a community effort that this evaluation harness is built to support.

The revision also adds a generative contribution that is structurally unavailable in other methods (such as NEMO and PhysMAP). HIPPIE's conditional decoder enables cross-modal imputation (Figure 6B), cross-species counterfactual decoding (Figure 6A; macaque cerebellar neurons decoded under the mouse source label), linear probing of cell-type identity from a single shared latent space without fine-tuning (Figure 6C; BA 0.64 mouse / 0.63 macaque from one checkpoint), and latent interpolation producing smooth morphological transitions between species (Figure 6D) and recording technologies (Supplementary cross-technology interpolation figures).

The issue of using ISI remains a concern. This may work for specific tasks and for specific species and brain areas. But the authors have acknowledged that. The only solution to this issue is to train the model on many variations of commonly used tasks and across many brain areas. The datasets used in this study provide proof of principle that the method works, but they are not representative and do not generalize across the field.

We agree that ISI generalizability remains a limitation and have noted this in the Limitations section of the Discussion. As described above, we have now included additional datasets, among them the two largest currently available in the field. We have also included a new data modality in the benchmarking (3D autocorrelogram), showing that the architecture is robust across input modalities. The cross-modal imputation experiments (Figure 6) further reveal that waveform and spike-time-derived metrics (ISI, autocorrelogram) capture different and complementary information that together improve the classification performance of HIPPIE.

The neuron shown in the answers to the reviewers' comments is atypical and appears to correspond to a recording near the neuron's axon. This is a concern since classification algorithms based on waveform features will classify this type of cell as a separate cluster. This is an issue with extracellular recordings: the position of the electrode relative to the neuron, and the neuron's morphology and orientation of its dendritic arbours and axon, influence the waveform. It would be useful to have a better characterization of the recordings and the layer in which they were recorded. I am not convinced that these datasets contain the necessary diversity. One possibility is to rule out positive and obviously biphasic waveforms. One may still argue that optogenetic tagging will address this problem, but, in my opinion, it does not unless the same sample size is used across different waveform types and electrode positions relative to neurons. I suspect these recordings usually target the grey matter, and they don't provide precise localization of each contact relative to that grey matter.

We appreciate this point and have addressed it quantitatively rather than treating optotagging alone as a sufficient answer. We classified every neuron's waveform as negative-going, positive-going, or biphasic (peak/trough amplitude ratio > 2: positive; < 0.5: negative; otherwise biphasic) and ran two analyses (See Methods; Subsection Waveform Polarity effect analysis; as well as Supplementary polarity tables).

Across datasets, the Pearson correlation between polarity composition and balanced accuracy was $|r| \leq 0.19$ for all polarity classes, indicating that no polarity fraction systematically predicts dataset difficulty. Within datasets, polarity-stratified accuracy gaps were inconsistent in direction across datasets, so no polarity class is universally easier or harder for HIPPIE to classify.

Interestingly, this polarity heterogeneity within a cell type offers a mechanistic explanation for the KNN-over-MLP advantage we observe on some datasets: when the same cell type occupies two distinct latent neighborhoods (one per polarity), KNN's local-neighborhood vote handles both without requiring them to be contiguous, while a global MLP decision boundary cannot. This argument is now discussed in the Discussion section.

Regarding waveform processing, multielectrode averages provide a more stable representation of waveform shape than max-amplitude single-electrode representations, at the cost of absolute amplitude magnitude. We acknowledge that this aggregation is not always practical, because the 2D geometry of a probe does not always match the 3D anatomy of the surrounding tissue; we observe this in the trimodal benchmark (macaque Lisberger) and in some cortical datasets, which further motivates multimodal integration. We see two natural paths forward: more optotagged data, so the model encounters enough instances of axon-near and other atypical waveforms to form stable subclusters, and improved waveform extraction through better spike-sorting pipelines. Both are included in the Discussion as natural follow-ups. HIPPIE's modular cVAE architecture accepts new feature branches without architectural changes, making the framework a substrate for these extensions rather than a finished product.

In conclusion, I believe this is a valuable contribution but needs extensive testing for validation in comparison to other methods.

We thank the reviewer for their guidance throughout this review process. The new version of the manuscript includes substantially more testing across species, brain areas, and input modalities, and we hope this increases the value of HIPPIE for the community.

Reviewer #1 (Remarks to the Author):

I thank the authors for their response. The revision addresses my baseline concerns well: NEMO and PhysMAP now receive the same nested cross-validation sweep, the shared evaluation harness standardizes splits and preprocessing, and hyperparameters are locked on a held-out dataset. The comparisons are far more credible as a result.

We thank the reviewer for the careful new read, and for acknowledging that the revised evaluation makes the comparisons substantially more credible.

With the corrected evaluation, however, the earlier shortcomings that inflated the original results now leave HIPPIE's classification performance essentially at parity with existing methods. The revised abstract accordingly softens the claim to "competitive with" state-of-the-art and shifts the emphasis to the generative decoder.

Under the corrected evaluation, HIPPIE's classification performance is on par with existing methods, and we have revised the abstract and main text to state this directly: the abstract now describes HIPPIE as classifying cell types "competitively with existing methods," and the Discussion notes that HIPPIE's advantage over the strongest baseline lies in its decoder rather than in classification rank.

But the generative capabilities are shown only as demonstrations, with no concrete use case in which they enable a task or improve on existing methods. As it stands, the classification claims are marginal and the generative contribution remains a capability without a demonstrated application.

We agree that the generative capabilities are currently a demonstration. We now state this directly in the limitations paragraph of the Discussion, noting that HIPPIE's generative capabilities are characterized here rather than deployed. Counterfactual decoding, cross-species interpolation, and cross-modal imputation behave as expected, but their value on a concrete downstream task remains untested.

Reviewer #3 (Remarks to the Author):

I am satisfied with the answers to my concerns. This is a dynamically evolving field, and in the absence of cell type tagging, this method is a valuable contribution.

We thank the reviewer for the positive assessment of our work.

Reviewer #3 (Remarks on code availability):
NA

N/A
